# Association of tyrosine kinase 2 polymorphisms with susceptibility to microscopic polyangiitis in a Guangxi population

**DOI:** 10.7717/peerj.18735

**Published:** 2024-12-23

**Authors:** Binglan Yang, Liepeng Chu, Fei Feng, Shurong Lu, Chao Xue

**Affiliations:** Department of Nephrology, The Second Affiliated Hospital of Guangxi Medical University, Nanning, Guangxi, China

**Keywords:** Microscopic polyangiitis, TYK2, Polymorphism, Susceptibility

## Abstract

**Background:**

Heredity and epigenetics affect the pathogenesis of microscopic polyangiitis (MPA). Tyrosine kinase 2 (TYK2) polymorphisms (rs2304256C > A, rs280519A > G, and rs12720270G > A) may be potential protective factors against anti-neutrophil cytoplasmic antibody (ANCA)-associated vasculitis (AAV). Current research suggests that TYK2 is associated with various autoimmune diseases; however, no study has examined the relationship between TYK2 polymorphisms and AAV. This study assessed the effect of TYK2 polymorphisms on susceptibility to MPA.

**Methods:**

Overall, 562 Chinese participants (265 patients with MPA and 297 healthy volunteers) were recruited. Polymerase chain reactions combined with high-throughput sequencing were used to analyze polymorphic loci, while logistic regression analysis was used to assess the relationship between polymorphism of the TYK2 gene and MPA susceptibility.

**Results:**

In males, individuals with the CA genotype (rs2304256) in the overdominant model showed a significantly reduced risk of MPA (odds ratio (OR) = 0.52; 95% confidence interval (CI) [0.29–0.93]; *p* = 0.025). Regarding rs280519, male carriers of the AG genotype had a significantly lower risk of developing MPA in both the codominant (OR = 0.51; 95% CI [0.28–0.93]; *p* = 0.039) and overdominant (OR = 0.48; 95% CI [0.27–0.86]; *p* = 0.013) models. The GA genotype of rs12720270 was associated with low susceptibility to MPA in males (OR = 0.52; 95% CI [0.29–0.93]; *p* = 0.027).

**Conclusions:**

This study indicates that mutations in the TYK2 gene (rs2304256, rs280519, and rs12720270) may be associated with a reduced risk of MPA in the male Chinese population in Guangxi. The A allele of single nucleotide polymorphism (SNP) rs2304256 may be a protective factor against MPA, while the G alleles of SNPs rs280519 and rs12720270 are protective factors against MPA.

## Introduction

Anti-neutrophil cytoplasmic antibody (ANCA)-associated vasculitis (AAV) is a heterogeneous group of small vasculitis of unknown etiology, characterized by the presence of proteinase 3 (PR3) and myeloperoxidase (MPO) in the serum (Kitching et al., 2020). This condition frequently involves inflammation and necrosis of the vessel wall. There are several types of this systemic vasculitis, including granulomatosis with polyangiitis (GPA) (Puechal, 2020), microscopic polyangiitis (MPA) (Aiyegbusi et al., 2021; Hunter et al., 2020), and eosinophilic granulomatosis with polyangiitis (EGPA) (Treccani et al., 2024). The distribution of AAV subtypes varies by region. GPA is prevalent in Northern Europe (Berti et al., 2017), while MPA is more common in China and Japan (Fujimoto et al., 2011; Watts & Scott, 2012).

The incidence of MPA ranges from 0.5 to 24.0 cases per million person-years, and its prevalence ranges from 9.0 to 94.0 cases per million person-years, with onset ages ranging from 55 to 75 years (Kitching et al., 2020). In Asia, the reported prevalence of AAV is between 46 and 421 per million population (Kawasaki & Tsuchiya, 2021). Kidney damage occurs in nearly all patients with MPA, characterized by necrotizing and crescentic pauci-immune glomerulonephritis. The deterioration of renal function can lead to end-stage renal disease and increased mortality (Binda, Moroni & Messa, 2018).

The human leukocyte antigen (HLA) region is a significant genetic risk factor for AAV (Trivioli et al., 2022). Genome-wide studies have identified a unique association between MPA and HLA-DQ (Li et al., 2021). In Chinese populations, the alleles HLA DQA1*03:02 and DQB1*03:03 are associated with MPO-AAV susceptibility (Wang et al., 2019). Similarly, in Japanese populations, these alleles are associated with both MPO-AAV susceptibility and recurrence risk (Kawasaki et al., 2023). The genetic studies enhance our understanding of the genetic susceptibility factors contributing to AVV, thereby improving patient management and treatment strategies.

Tyrosine kinase 2 (TYK2) is a vital signal transduction kinase in Janus kinase (JAK)/signal transducer and activator of transcription signaling pathway (Gonciarz et al., 2021), which is crucial in differentiating T helper 1 (Th1) and T helper type 17 (Th17) cells. Dysfunction of TYK2 can contribute to the development of autoimmune and inflammatory diseases (Muromoto et al., 2021). The necessity of TYK2 activity for interleukin (IL)-12, IL-23, and type I interferon (IFN1) signaling has been demonstrated experimentally in mice with TYK2 deficiency (Gorman et al., 2019). Moreover, inhibiting TYK2 activity has been reported to block the downstream signal transduction of IL-12 and other cytokines (Elyoussfi et al., 2023).

TYK2 gene mutations are genetically linked to ankylosing spondylitis, psoriasis, Crohn’s disease, ulcerative colitis, type 1 diabetes, multiple sclerosis (MS), lupus erythematosus (SLE), and rheumatoid arthritis (RA) (Parkes et al., 2013). Currently, research indicates that multiple selective inhibitors of TYK2 have been approved for treating autoimmune diseases, such as plaque psoriasis (Rusinol & Puig, 2023; Yuan et al., 2023). Novel TYK2 inhibitors are expected to have significant clinical impacts (Muromoto et al., 2021). Previous research has reported a close association between TYK2 variants rs34536443 and AAV in European individuals (Ortiz-Fernandez et al., 2020); however, no reports currently exist on the association between TYK2 gene rs2304256, rs280519, rs1272027 variants and patients with MPA.

## Materials and Methods

### Ethics approval

This study was approved by the Medical Ethics Committee of The Second Affiliated Hospital of Guangxi Medical University (2018 KY-0100) and was conducted in compliance with the Declaration of Helsinki. We have obtained written consent from all subjects.

### Study subjects

The MPA cohort comprised 265 patients with MPA from the Second Affiliated Hospital of Guangxi Medical University between January 2005 and January 2019, whose diagnosis criteria met the International Chapel Hill Consensus Conference Nomenclature of Vasculitides criteria (Jennette et al., 2013). Patients with secondary vasculitis caused by other factors (external infections, tumors, and drugs) and with other autoimmune diseases (RA, SLE, and Henoch-Schonlein purpura) were excluded. In addition, 297 healthy subjects who were confirmed to be free of MPA, other autoimmune diseases, and malignancies were included in the normal control group.

### Genotyping and data quality control

Genotyping was performed using multiplex polymerase chain reaction (PCR) and high-throughput sequencing (Sangon Biotech, Shanghai, China). **Data quality control:** First, any part of the sequence containing the sequenced splitter sequence was excised using cutadapt (v 1.2.1) (Martin, 2011) software; subsequently, the remaining sequence was quality-controlled using PRINSEQ-lite (v 0.20.3) software (Huang et al., 2018), and bases with a quality threshold <20 were removed from the 3′ end to the 5′ end of the sequence. The remaining sequences were considered to be qualified for quality control. **Genotyping:** The mapping program BWA-MEM (v 0.7.13-r1126) (Alganmi & Abusamra, 2023) was used to align the qualified sequences to the reference genome (Genome Reference Consortium Human Build 37). According to the comparison results (Zheng-Bradley et al., 2017), the genotypic results of the target locus were calculated using samtools software (version 0.1.18) (Li et al., 2009a). Finally, Annovar (version 2018-04-16) software (Wang, Li & Hakonarson, 2010) was used to annotate the mutation sites. Genomic DNA was extracted using the corresponding kit (cat. no. DP319‑02; Beijing Tiangen Biotech Co., Ltd., Beijing, China). A Nanodrop 2000 spectrophotometer (Thermo Fisher Scientific, Inc., Watham, MA, USA) was used to measure the concentration of DNA.

### Statistical analysis

Statistical analyses were performed using IBM SPSS Statistics for Windows, version 26.0 (IBM Corp., Armonk, NY, USA) (Liang, Fu & Wang, 2019). We used the two-sample T-test and Chi-square test to analyze the demographic features of the subjects. The Chi-square and Fisher’s exact tests were used to evaluate Hardy–Weinberg equilibrium, genotype, and allele frequencies. SNPstats (https://www.snpstats.net/start.htm) (Sole et al., 2006), a web tool used for SNP analysis, was utilized to examine the link between TYK2 gene polymorphism and MPA susceptibility with an unmatched case-control design and unconditional logistic regression. Indeed, to assess the association between TYK2 gene polymorphisms and MPA susceptibility, odds ratios (ORs) and 95% confidence intervals (CIs) were calculated after adjustment for age, race, and gender. In addition, haplotype analysis (Li et al., 2009b) was estimated using SHEsis online software (Shi & He, 2005). Bonferroni correction was performed for multiple analyses with more than two groups. *p*-value < 0.05 was considered significant.

## Results

### Characteristics of the study subjects

The case group comprised 265 cases, including 96 males and 169 females, with 163 and 102 Han and non-Han Chinese, respectively. The participant’s ages ranged from 18 to 86 years, with a median age of 59 years. The control group comprised 297 healthy volunteers who underwent physical examinations during the same period, including 124 males and 173 females, with 220 and 77 Han and non-Han Chinese, respectively. The age range was between 18 and 81 years, with a median age of 48 years. While no significant difference was found in the gender distribution between the two groups (*p* = 0.18), significant differences were observed in the ethnicity and age distribution (*p* < 0.001). Further stratification analysis using 60 years as the cutoff point showed no statistically significant difference between the two groups (Age < 60, *p* = 0.099; Age ≥ 60, *p* = 0.621). Theoretically, the control group’s age and gender should match those of the patients in the MPA group; however, only partial matching was observed between the case and control groups. Consequently, in the subsequent genetic model and subgroup analyses, adjustment was made for confounding factors, including age and ethnicity (Table 1).

**Table 1:** Demographic features of MPA cases and control group.

Variable	Case (*n* = 265)	Control (*n* = 297)	*p*
Gender (male/female)	96/169	124/173	0.18
Ethnicity (Han/non-Han)	163/102	220/77	<0.01*
Age (years)	59 (47, 67)^a^	48 (38, 55.5)^a^	<0.01*
Age < 60 (years)	136	244	0.099
Age ≥ 60 (years)	129	53	0.621

**Notes:**

MPA, microscopic polyangiitis; n, number of people.

a The description of skewed distribution data statistics uses median (lower quartile, upper quartile) representation; *p*-value, the Student’s t-test or the Chi-square test was used to compare variables in groups.

* Denotes statistical significance (*p* < 0.05).

### Information for selected single nucleotide polymorphisms (SNPs)

Table 2 presents the basic information of four TYK2 SNP loci and the preliminary analysis of susceptibility to MPA. The four SNPs selected in this study were located in different regions of the TYK2 gene on chromosome 19, including exons (rs2304256 and rs2304255) and introns (rs280519 and rs12720270). The genotype frequency distributions of all SNPs were in Hardy–Weinberg equilibrium (*p* > 0.05), and minor allele frequency (MAF) was also calculated for patients with MPA and healthy controls. Previous studies have demonstrated that rare genotypes may produce spurious findings (Tabangin, Woo & Martin, 2009). Therefore, we prioritized SNPs with MAF > 0.05 as the primary research targets (The International HapMap Consortium, 2003). However, rs2304255 did not meet the MAF threshold (MAF ≤ 5%), showing no statistical significance; thus, it was excluded from subsequent correlation analyses.

**Table 2:** Basic information of the selected SNPs.

SNP	Chromosome	Position	Alleles	Gene	Location	MAF		HWE-*p*	Allele-*p*	Genotype-*p*
						Control	Case			
rs2304256	chr19	10,475,652	C > A	TYK2	Exon 8	0.40	0.38	0.46	0.55	0.83
rs280519	chr19	10,472,933	A > G	TYK2	Intron 11	0.33	0.33	0.19	0.91	0.65
rs2304255	chr19	10,475,649	C > T	TYK2	Exon 8	0.03	0.03	0.61	—	—
rs12720270	chr19	10,475,760	G > A	TYK2	Intron 7	0.43	0.42	0.34	0.74	0.89

**Note:**

MPA, microscopic polyangiitis; SNP, single nucleotide polymorphism; MAF, minor allele frequency; HWE, Hardy-Weinberg equilibrium; chr, chromosome; The *p*-value was calculated using Chi-square test and Fisher’s exact test; Bonferroni correction was used for multiple comparison.

### Association between the selected SNPs and MPA susceptibility

We conducted a further analysis across different layers utilizing various genetic models to assess the impact of TYK2 variant polymorphisms (rs2304256, rs280519, and rs12720270) on the risk of MPA. However, no statistically significant differences were observed (see Table 3). Consequently, we performed a subgroup analysis of the sample based on gender (male and female), age (<60 years and ≥60 years), and ethnicity (Han and non-Han). The results showed that the association between SNPs and MPA risk was influenced by sex, particularly in the male subgroup (*p* < 0.05). Specifically, rs2304256 reduced the risk of MPA with the CA genotype in the overdominant model (OR = 0.52; 95% CI [0.29–0.93]; *p* = 0.025). In rs280519, carriers of the AG genotype in the codominant (OR = 0.51; 95% CI [0.28–0.93]; *p* = 0.039) and overdominant models (OR = 0.48; 95% CI [0.27–0.86]; *p* = 0.013) showed a significantly lower risk of MPA. For rs12720270, the GA genotype in the overdominant model was associated with a low susceptibility to MPA (OR = 0.52; 95% CI [0.29–0.93]; *p* = 0.027) (Table 4). However, no positive results were observed in the female subgroup (Table 5).

**Table 3:** The relationship between the SNPs and the risk of MPA in Guangxi population in different genetic models.

SNP	Models	Genotype/Allele	Control	Case	OR (95% CI)	*p*‑value
rs2304256	Allele	A	355 (59.8%)	326 (61.5%)	1.08 [0.85–1.37]	0.55
		C	239(40.2%)	204 (38.5%)		
	Codominant	AA	103 (34.7%)	98 (37%)	1.00	0.79
		CA	149 (50.2%)	130 (49.1%)	0.87 [0.60–1.29]	
		CC	45 (15.2%)	37 (14%)	0.95 [0.55–1.65]	
	Dominant	AA	103 (34.7%)	98 (37%)	1.00	0.54
		CA-CC	194 (65.3%)	167 (63%)	0.89 [0.62–1.28]	
	Recessive	AA-CA	252 (84.8%)	228 (86%)	1.00	0.92
		CC	45 (15.2%)	37 (14%)	1.03 [0.62–1.70]	
	Overdominant	AA-CC	148 (49.8%)	135 (50.9%)	1.00	0.51
		CA	149 (50.2%)	130 (49.1%)	0.89 [0.62–1.26]	
rs280519	Allele	G	396 (66.7%)	355 (67.0%)	0.99 [0.77–1.26]	0.91
		A	198 (33.3%)	175 (33.0%)		
	Codominant	GG	127 (42.8%)	119 (44.9%)	1.00	0.37
		AG	142 (47.8%)	117 (44.1%)	0.82 [0.57–1.20]	
		AA	28 (9.4%)	29 (10.9%)	1.22 [0.66–2.28]	
	Dominant	GG	127 (42.8%)	119 (44.9%)	1.00	0.5
		AG-AA	170 (57.2%)	146 (55.1%)	0.88 [0.62–1.26]	
	Recessive	GG-AG	269 (90.6%)	236 (89.1%)	1.00	0.32
		AA	28 (9.4%)	29 (10.9%)	1.35 [0.74–2.44]	
	Overdominant	GG-AA	155 (52.2%)	148 (55.9%)	1.00	0.2
		AG	142 (47.8%)	117 (44.1%)	0.79 [0.56–1.13]	
rs12720270	Allele	A	336 (56.6%)	305 (57.5%)	1.04 [0.82–1.32]	0.74
		G	258 (43.4%)	225 (42.5%)		
	Codominant	AA	91 (30.6%)	86 (32.5%)	1.00	0.63
		GA	154 (51.9%)	133 (50.2%)	0.83 [0.56–1.24]	
		GG	52 (17.5%)	46 (17.4%)	0.97 [0.57–1.66]	
	Dominant	AA	91 (30.6%)	86 (32.5%)	1.00	0.46
		GA-GG	206 (69.4%)	179 (67.5%)	0.87 [0.59–1.26]	
	Recessive	AA-GA	245 (82.5%)	219 (82.6%)	1.00	0.73
		GG	52 (17.5%)	46 (17.4%)	1.09 [0.68–1.74]	
	Overdominant	AA-GG	143 (48.1%)	132 (49.8%)	1.00	0.34
		GA	154 (51.9%)	133 (50.2%)	0.84 [0.59–1.20]	

**Notes:**

MPA, microscopic polyangiitis; SNP, single nucleotide polymorphism; OR, odds ratio; CI, confidence interval; The *p*-value, OR, and 95% CI were derived from a logistic regression model adjusted for age, ethnicity, and gender.

**Table 4:** The relationship between the SNPs and the risk of MPA in male of Guangxi population in different genetic models.

SNP	Models	Genotype/Allele	Control	Case	OR (95% CI)	*p*-value
rs2304256	Allele	A	140 (56.4%)	124 (64.6%)	0.711 [0.48–1.05]	0.085
		C	108 (43.5%)	68 (35.4%)		
	Codominant	AA	36 (29%)	43 (44.8%)	1.00	0.082
		CA	68 (54.8%)	38 (39.6%)	0.53 [0.28–0.99]	
		CC	20 (16.1%)	15 (15.6%)	1.05 [0.43–2.54]	
	Dominant	AA	36 (29%)	43 (44.8%)	1.00	0.11
		CA-CC	88 (71%)	53 (55.2%)	0.62 [0.34–1.12]	
	Recessive	AA-CA	104 (83.9%)	81 (84.4%)	1.00	0.31
		CC	20 (16.1%)	15 (15.6%)	1.52 [0.67–3.42]	
	Overdominant	AA-CC	56 (45.2%)	58 (60.4%)	1.00	0.025*
		CA	68 (54.8%)	38 (39.6%)	0.52 [0.29–0.93]	
rs280519	Allele	G	155 (62.5%)	134 (69.8%)	1.59 [1.06–2.39]	0.11
		A	93 (37.5%)	58 (31.2%)		
	Codominant	GG	43 (34.7%)	49 (51%)	1.00	0.039*
		AG	69 (55.6%)	36 (37.5%)	0.51 [0.28–0.93]	
		AA	12 (9.7%)	11 (11.5%)	1.30 [0.47–3.57]	
	Dominant	GG	43 (34.7%)	49 (51%)	1.00	0.078
		AG-AA	81 (65.3%)	47 (49%)	0.60 [0.33–1.06]	
	Recessive	GG-AG	112 (90.3%)	85 (88.5%)	1.00	0.2
		AA	12 (9.7%)	11 (11.5%)	1.88 [0.72–4.91]	
	Overdominant	GG-AA	55 (44.4%)	60 (62.5%)	1.00	0.013*
		AG	69 (55.6%)	36 (37.5%)	0.48 [0.27–0.86]	
rs12720270	Allele	A	135 (54.4%)	115 (59.9%)	0.80 [0.55–1.17]	0.25
		G	113 (45.6%)	77 (40.1%)		
	Codominant	AA	33 (26.6%)	37 (38.5%)	1.00	0.071
		GA	69 (55.6%)	41 (42.7%)	0.57 [0.30–1.09]	
		GG	22 (17.7%)	18 (18.8%)	1.32 [0.55–3.14]	
	Dominant	AA	33 (26.6%)	37 (38.5%)	1.00	0.25
		GA-GG	91 (73.4%)	59 (61.5%)	0.70 [0.38–1.28]	
	Recessive	AA-GA	102 (82.3%)	78 (81.2%)	1.00	0.12
		GG	22 (17.7%)	18 (18.8%)	1.85 [0.85–4.03]	
	Overdominant	AA-GG	55 (44.4%)	55 (57.3%)	1.00	0.027*
		GA	69 (55.6%)	41 (42.7%)	0.52 [0.29–0.93]	

**Notes:**

MPA, microscopic polyangiitis; SNP, single nucleotide polymorphism; OR, odds ratio; CI, confidence interval; The *p*-value, OR, and 95% CI were derived from a logistic regression model adjusted for age and ethnicity.

* Denotes statistical significance (*p* < 0.05).

**Table 5:** The relationship between the SNPs and the risk of MPA in female of Guangxi population in different genetic models.

SNP	Models	Genotype/Allele	Control	Case	OR (95% CI)	*p*‑value
rs2304256	Allele	A	215 (62.1%)	201 (59.6%)	1.11 [0.82–1.51]	0.50
		C	131 (37.9%)	136 (40.4%)		
	Codominant	AA	67 (38.7%)	55 (32.5%)	1.00	0.6
		CA	81 (46.8%)	92 (54.4%)	1.22 [0.74–1.99]	
		CC	25 (14.4%)	22 (13%)	0.91 [0.44–1.86]	
	Dominant	AA	67 (38.7%)	55 (32.5%)	1.00	0.58
		CA-CC	106 (61.3%)	114 (67.5%)	1.14 [0.71–1.83]	
	Recessive	AA-CA	148 (85.5%)	147 (87%)	1.00	0.53
		CC	25 (14.4%)	22 (13%)	0.81 [0.42–1.56]	
	Overdominant	AA-CC	92 (53.2%)	77 (45.6%)	1.00	0.33
		CA	81 (46.8%)	92 (54.4%)	1.25 [0.80–1.96]	
rs280519	Allele	G	241 (69.7%)	221 (65.4%)	1.22 [0.88–1.67]	0.23
		A	105 (30.3%)	117 (34.6%)		
	Codominant	GG	84 (48.5%)	70 (41.4%)	1.00	0.83
		AG	73 (42.2%)	81 (47.9%)	1.14 [0.71–1.83]	
		AA	16 (9.3%)	18 (10.7%)	1.20 [0.54–2.65]	
	Dominant	GG	84 (48.5%)	70 (41.4%)	1.00	0.56
		AG-AA	89 (51.5%)	99 (58.6%)	1.15 [0.73–1.81]	
	Recessive	GG-AG	157 (90.8%)	151 (89.3%)	1.00	0.76
		AA	16 (9.2%)	18 (10.7%)	1.12 [0.53–2.40]	
	Overdominant	GG-AA	100 (57.8%)	88 (52.1%)	1.00	0.68
		AG	73 (42.2%)	81 (47.9%)	1.10 [0.70–1.73]	
rs12720270	Allele	A	201 (58.1%)	190 (56.2%)	1.08 [0.80–1.46]	0.62
		G	145 (41.9%)	148 (43.8%)		
	Codominant	AA	58 (33.5%)	49 (29%)	1.00	0.76
		GA	85 (49.1%)	92 (54.4%)	1.08 [0.64–1.79]	
		GG	30 (17.3%)	28 (16.6%)	0.85 [0.43–1.69]	
	Dominant	AA	58 (33.5%)	49 (29%)	1.00	0.95
		GA-GG	115 (66.5%)	120 (71%)	1.02 [0.62–1.65]	
	Recessive	AA-GA	143 (82.7%)	141 (83.4%)	1.00	0.5
		GG	30 (17.3%)	28 (16.6%)	0.81 [0.44–1.49]	
	Overdominant	AA-GG	88 (50.9%)	77 (45.6%)	1.00	0.57
		GA	85 (49.1%)	92 (54.4%)	1.14 [0.72–1.79]	

**Notes:**

MPA, microscopic polyangiitis; SNP, single nucleotide polymorphism; OR, odds ratio; CI, confidence interval; The *p*-value, OR, and 95% CI were derived from a logistic regression model adjusted for age and ethnicity.

Similarly, we performed a subgroup analysis based on age (<60 years and ≥60 years) and ethnicity (Han and non-Han) within the sample; however, no statistically significant differences were observed (*p* > 0.05) (Tables S1 and S2).

### Correlation of the haplotypes of TYK2 gene with MPA susceptibility

The analysis of haplotypes of the TYK2 gene in relation to MPA susceptibility is presented in Table 6. Four haplotypes, formed by the alleles of rs2304256, rs280519, and rs12720270, were identified. However, these haplotypes did not demonstrate any significant association with susceptibility to MPA.

**Table 6:** Correlations between the haplotypes of TYK2 gene and MPA susceptibility.

Geng	SNP	Haplotypes	Control	Case	χ^2^	*p*	OR (95% CI)
TYK2	rs2304256/rs280519/rs12720270	AGA	336.00 (0.566)	303.94 (0.573)	0.092	0.762	1.037 [0.819–1.314]
		AGG	19.00 (0.032)	21.00 (0.040)	0.485	0.486	1.251 [0.665–2.354]
		CAG	198.00 (0.333)	173.94 (0.328)	0.025	0.873	0.980 [0.764–1.257]
		CGG	41.00 (0.069)	30.06 (0.057)	0.701	0.402	0.813 [0.500–1.321]

**Note:**

SNP, single nucleotide polymorphism; OR, odds ratio; CI, confidence interval; The *p*-value was calculated using Chi-square test and Fisher’s exact test; Bonferroni correction was used for multiple comparison.

## Discussion

Current research indicates that MPA is closely associated with genetic factors, environmental influences, and patient age (Hunter et al., 2020). In this case-control study, we examined the relationships between three potential effect SNPs of the TYK2 gene and MPA risk in a Guangxi population in China. TYK2 is the first member of the JAK family, a 27.9-kb gene with 25 exons located on chromosome 19p13.2 (Lindqvist et al., 2000). This gene moderates immune responses to IL-12, IL-23, IFNα, and Th17 (Gonzalez Lopez de Turiso & Guckian, 2022). Research has shown that IL-12, IL-23, IFNα, Th1, and Th17 are closely associated with MPA (Ortiz-Fernandez et al., 2020; Yuan et al., 2023). For example, in an ANCA-mediated mouse model of experimental vasculitis, Hoshino et al. (2008) found that activated neutrophils produced IL-17A and IL-23 *via* the classical complement pathway in response to MPO-ANCA. This pathway provides a local environment that promotes IL-17 production and Th17-mediated autoimmunity, becoming the first step in initiating chronic autoimmune inflammation (Hoshino et al., 2008).

Rs2304256 is one of the most common SNPs of TYK2, located in exon 8, and its genetic association has been widely investigated (Gonciarz et al., 2021; Morand et al., 2024). Although rs2304256 is associated with SLE in Finnish, Swedish, British (Cunninghame Graham et al., 2007; Hellquist et al., 2009; Sigurdsson et al., 2005; Suarez-Gestal et al., 2009), and Chinese Han (Tang et al., 2015) populations, it shows reversed association in Hong Kong (Li et al., 2011) and Japanese (Kyogoku et al., 2009) populations. TYK2 rs2304256 was verified to be closely related to systemic sclerosis susceptibility (SSc) in the European population (Lopez-Isac et al., 2016), while the results were negative in the Chinese Han SSc population (Liu et al., 2021). These inconsistencies appear to arise from differences in geography and ethnicity (Liu et al., 2021). Pellenz et al. (2021) pooled data from 34 articles for a meta-analysis assessing the impact of multiple TYK2 variants on susceptibility to autoimmune diseases. Their data suggested that rs2304256 is a susceptibility factor for several autoimmune diseases, including SLE, MS, and rheumatoid RA, with the A allele acting as a protective factor (Pellenz et al., 2021). Our study illustrated that the rs2304256 (C > A) variant in the overdominant model potentially decreases susceptibility to MPA in males. This effect is because the A allele contributes to the substitution of valine 362 with phenylalanine in the JAK homologous four region. This domain is crucial for the interaction between TYK2 and IFNAR1, which is essential for maintaining IFNAR1 expression on the cell membrane. The amino acid substitution affects the processing of precursor mRNA (Li et al., 2020), leading to the downregulation of IFN-α signaling and subsequent reduction in proinflammatory cytokines and inflammation (Marroqui et al., 2015). Additionally, both the PolyPhen-2 and sorting intolerant from tolerant tools indicate that rs2304256 is a benign variant (Adzhubei et al., 2010; Kumar, Henikoff & Ng, 2009), aligning with our research findings.

The rs280519 and rs12720270 variants are located in the TYK2 intron region. An earlier meta-analysis, including 16,335 patients with SLE and 30,065 controls, revealed that the rs280519 polymorphism was significantly associated with SLE risk in Caucasians and Asians (Lee & Bae, 2016). A Turkish study involving 60 patients with Crohn’s disease and 151 patients with ulcerative colitis found that the rs280519 AA genotype was a risk factor for ulcerative colitis, while the AG genotype was a protective factor for ulcerative colitis and Crohn’s disease (Can et al., 2015). Affected by the IFN signaling pathway, the rs280519 (A > G) G allele may influence the severe National Institute on Aging classification in patients with chronic hepatitis C (Lopez-Rodriguez et al., 2017). Moreover, both the rs280519 and rs12720270 variants reduced the risk of juvenile idiopathic arthritis in the Chinese Han population (Qian et al., 2022). Additionally, rs12720270 may downregulate coronavirus disease 2019 severity by decreasing TYK2 expression (Zabihi Rizi et al., 2023). Our study results showed that rs280519A > G might be related to autoimmune and chronic inflammatory diseases, which is consistent with previous research findings. Similarly, in the codominant and overdominant models, the AG genotype of rs280519 can reduce male susceptibility to MPA. In the overdominant model, the GA genotype of rs12720270 can also reduce male susceptibility to MPA. We hypothesize that the intronic variants rs280519 and rs12720270 may cause splicing abnormalities, intronic mutations, protein-coding disruption, altering of residue positions, and loss or insertion of the internal coding frame (Bryen et al., 2019). This may lead to abnormal expression of TYK2, thereby affecting the cytokine signaling pathway (Zabihi Rizi et al., 2023) and ultimately reducing susceptibility to MPA. However, no significant effects were found in females across the three loci (Table 4). Overall, this study explored the relationship between TYK2 gene polymorphisms in the Guangxi population and susceptibility to MPA. It revealed that TYK2 gene polymorphisms rs2304256, rs280519, and rs12720270 in the male population of Guangxi may be associated with susceptibility to MPA. The strength of this study include the subgroup analysis across various genetic models and multiple SNP interactions. However, as a single-center retrospective study with limited cases, these conclusions require validation through larger, multi-center, prospective clinical studies.

## Conclusions

This study found that mutations in the TYK2 gene—rs2304256, rs280519, and rs12720270—may be associated with a reduced risk of MPA in the male Chinese population in Guangxi. The A allele of SNP rs2304256 may be a protective factor against MPA, while the G alleles of SNPs rs280519 and rs12720270 are protective factors against MPA. However, the potential molecular mechanisms require further investigation. For example, we can use gene editing technology to study the effect of TYK2 gene mutations on cell function and animal phenotypes to better understand their role in the pathogenesis of MPA and provide a valuable theoretical basis for its treatment.

## Supplemental Information

10.7717/peerj.18735/supp-1 Supplemental Information 1 Raw data.

10.7717/peerj.18735/supp-2 Supplemental Information 2 The relationship between the SNPs and the risk of MPA in in different ethnicity groups in Guangxi in different genetic models.

10.7717/peerj.18735/supp-3 Supplemental Information 3 Association between the SNPs and the risk of MPA in different age groups in Guangxi in different genetic models.

10.7717/peerj.18735/supp-4 Supplemental Information 4 Statistical results for Tables 2 and 6.

10.7717/peerj.18735/supp-5 Supplemental Information 5 Statistical results for Table 3.

10.7717/peerj.18735/supp-6 Supplemental Information 6 Statistical results for Table 4, male.

10.7717/peerj.18735/supp-7 Supplemental Information 7 Statistical results for Table 5, female.

10.7717/peerj.18735/supp-8 Supplemental Information 8 Statistical results for Table S1, Han.

10.7717/peerj.18735/supp-9 Supplemental Information 9 Statistical results for Table S1, nonHan.

10.7717/peerj.18735/supp-10 Supplemental Information 10 Statistical results for Table S2, Age more than 60.

10.7717/peerj.18735/supp-11 Supplemental Information 11 Statistical results for Table S2, Age less than 60.

## Additional Information and Declarations

### Data Availability

The following information was supplied regarding data availability: The raw measurements are available in the supplemental Files 1 and 2.
