# Supplementary material for: Association of tyrosine kinase 2 polymorphisms with susceptibility to microscopic polyangiitis in a Guangxi population
Source: PeerJ. 2024 Dec 23;12:e18735. doi: 10.7717/peerj.18735 (PMC11670758; doi:10.7717/peerj.18735)
Supplement: Supplemental Information 2 [file peerj-12-18735-s002.docx]

Supplementary Table 1: The relationship between the SNPs and the risk of MPA in in different ethnicity groups in Guangxi in different genetic models

| **SNP** | **Models** | **Genotype**  **/Allele** | | | **Ethnicity=Han** | | | **Ethnicity =Non-Han** | | | | | | | | | | |
| --- | --- | --- | --- | --- | --- | --- | --- | --- | --- | --- | --- | --- | --- | --- | --- | --- | --- | --- |
|  |  |  |  |  | Control | Case | OR (95% CI) | | *P*‑value | |  | Control Case | | |  | | OR (95% CI) | *P*‑value |
| rs2304256 | Allele | | A | 264 | | 198 | 0.97(0.72-1.3) | | 0.84 |  | | | 91 | 128 | | 0.86(0.56-1.32) | | 0.48 |
|  |  |  | C | 176 | | 128 |  |  |  |  |  |  | 63 | 76 | |  |  |  |
|  | Codominant | | AA | 75 | | 60 | 1.00 | | 0.53 | | | | 28 | 38 | | 1.00 | | 0.45 |
|  |  |  | CA | 114 | | 78 | 0.87 (0.55-1.39) | |  |  |  |  | 35 | 52 | | 0.88 (0.44-1.76) | |  |
|  |  |  | CC | 31 | | 25 | 1.25 (0.64-2.46) | |  |  |  |  | 14 | 12 | | 0.54 (0.20-1.43) | |  |
|  | Dominant | | AA | 75 | | 60 | 1.00 | | 0.8 | | | | 28 | 38 | | 1.00 | | 0.47 |
|  |  |  | CA-CC | 145 | | 103 | 0.94 (0.60-1.47) | |  |  |  |  | 49 | 64 | | 0.79 (0.41-1.52) | |  |
|  | Recessive | | AA-CA | 189 | | 138 | 1.00 | | 0.33 | | | | 63 | 90 | | 1.00 | | 0.23 |
|  |  |  | CC | 31 | | 25 | 1.36 (0.73-2.52) | |  |  |  |  | 14 | 12 | | 0.58 (0.24-1.41) | |  |
|  | Overdominant | | AA-CC | 106 | | 85 | 1.00 | | 0.36 | | | | 42 | 50 | | 1.00 | | 0.87 |
|  |  |  | CA | 114 | | 78 | 0.82 (0.54-1.26) | |  |  |  |  | 35 | 52 | | 1.06 (0.56-1.98) | |  |
| rs280519 | Allele | | G | 291 | | 216 | 0.99(0.74-1.35) | | 0.97 | | | | 105 | 139 | | 1.00(0.64-1.57) | | 0.99 |
|  |  | | A | 149 | | 110 |  |  |  |  |  |  | 49 | 65 | |  |  |  |
|  | Codominant | | GG | 93 | | 74 | 1.00 | | 0.32 | | | | 34 | 45 | | 1.00 | | 0.82 |
|  |  | | AG | 105 | | 68 | 0.82 (0.52-1.30) | |  | | | | 37 | 49 | | 0.82 (0.42-1.59) | |  |
|  |  | | AA | 22 | | 21 | 1.41 (0.69-2.90) | |  | | | | 6 | 8 | | 0.80 (0.23-2.71) | |  |
|  | Dominant | | GG | 93 | | 74 | 1.00 | | 0.69 | | | | 34 | 45 | | 1.00 | | 0.54 |
|  |  | | AG-AA | 127 | | 89 | 0.92 (0.60-1.41) | |  | | | | 43 | 57 | | 0.82 (0.43-1.55) | |  |
|  | Recessive | | GG-AG | 198 | | 142 | 1.00 | | 0.21 | | | | 71 | 94 | | 1.00 | | 0.84 |
|  |  | | AA | 22 | | 21 | 1.55 (0.78-3.08) | |  | | | | 6 | 8 | | 0.89 (0.27-2.85) | |  |
|  | Overdominant | | GG-AA | 115 | | 95 | 1.00 | | 0.23 | | | | 40 | 53 | | 1.00 | | 0.61 |
|  |  | | AG | 105 | | 68 | 0.77 (0.50-1.18) | |  | | | | 37 | 49 | | 0.85 (0.45-1.60) | |  |
| rs12720270 | Allele | | A | 251 | | 188 | 0.98(0.73-1.30) | | 0.86 | | | | 85 | 117 | | 0.92(0.60-1.40) | | 0.68 |
|  |  | | G | 189 | | 138 |  |  |  |  |  |  | 69 | 87 | |  |  |  |
|  | Codominant | | AA | 68 | | 53 | 1.00 | | 0.7 | | | | 23 | 33 | | 1.00 | | 0.48 |
|  |  | | GA | 115 | | 82 | 0.92 (0.57-1.49) | |  | | | | 39 | 51 | | 0.66 (0.32-1.38) | |  |
|  |  | | GG | 37 | | 28 | 1.20 (0.62-2.29) | |  | | | | 15 | 18 | | 0.64 (0.25-1.62) | |  |
|  | Dominant | | AA | 68 | | 53 | 1.00 | | 0.94 | | | | 23 | 33 | | 1.00 | | 0.23 |
|  |  | | GA-GG | 152 | | 110 | 0.98 (0.62-1.55) | |  | | | | 54 | 69 | | 0.65 (0.33-1.31) | |  |
|  | Recessive | | AA-GA | 183 | | 135 | 1.00 | | 0.44 | | | | 62 | 84 | | 1.00 | | 0.64 |
|  |  | | GG | 37 | | 28 | 1.26 (0.70-2.24) | |  | | | | 15 | 18 | | 0.82 (0.36-1.85) | |  |
|  | Overdominant | | AA-GG | 105 | | 81 | 1.00 | | 0.52 | | | | 38 | 51 | | 1.00 | | 0.45 |
|  |  | | GA | 115 | | 82 | 0.87 (0.57-1.33) | |  | | | | 39 | 51 | | 0.78 (0.41-1.48) | |  |

NOTE:MPA: microscopic polyangiitis; SNP: single nucleotide polymorphism; OR: odds ratio; CI: confidence interval; The *p*-value, OR, and 95% CI were derived from a logistic regression model adjusted for age and ethnicity; * denotes statistical significance (*p*<0.05).
