# Supplementary material for: Association of tyrosine kinase 2 polymorphisms with susceptibility to microscopic polyangiitis in a Guangxi population
Source: PeerJ. 2024 Dec 23;12:e18735. doi: 10.7717/peerj.18735 (PMC11670758; doi:10.7717/peerj.18735)
Supplement: Supplemental Information 3 [file peerj-12-18735-s003.docx]

Supplementary Table 2: Association between the SNPs and the risk of MPA in different age groups in Guangxi in different genetic models

| **SNP** | **Models** | **Genotype**  **/Allele** | **Age (years)<60** | | | |  | | **Age (years)≥60** | | | |
| --- | --- | --- | --- | --- | --- | --- | --- | --- | --- | --- | --- | --- |
|  |  |  | Control | Case | OR (95% CI) | *P*‑value | |  | Control | Case | OR (95% CI) | *P*‑value |
| rs2304256 | Allele | A | 286 | 172 | 0.82(0.61-1.12) | 0.21 | | | 69 | 154 | 1.26(0.79-2.02) | 0.34 |
|  |  | C | 202 | 100 |  |  |  |  | 37 | 104 |  |  |
|  | Codominant | AA | 82 | 53 | 1.00 | 0.51 | | | 21 | 45 | 1.00 | 0.53 |
|  |  | CA | 122 | 66 | 0.87 (0.55-1.38) |  |  |  | 27 | 64 | 0.98 (0.48-2.00) |  |
|  |  | CC | 40 | 17 | 0.68 (0.35-1.32) |  |  |  | 5 | 20 | 1.78 (0.57-5.57) |  |
|  | Dominant | AA | 82 | 53 | 1.00 | 0.38 | | | 21 | 45 | 1.00 | 0.78 |
|  |  | CA-CC | 162 | 83 | 0.82 (0.53-1.28) |  |  |  | 32 | 84 | 1.10 (0.56-2.19) |  |
|  | Recessive | AA-CA | 204 | 119 | 1.00 | 0.32 | | | 48 | 109 | 1.00 | 0.26 |
|  |  | CC | 40 | 17 | 0.73 (0.40-1.36) |  |  |  | 5 | 20 | 1.81 (0.62-5.24) |  |
|  | Overdominant | AA-CC | 122 | 70 | 1.00 | 0.89 | | | 26 | 65 | 1.00 | 0.62 |
|  |  | CA | 122 | 66 | 0.97 (0.64-1.48) |  |  |  | 27 | 64 | 0.85 (0.43-1.65) |  |
| rs280519 | Allele | G | 322 | 189 | 0.85(0.62-1.17) | 0.32 | | | 74 | 166 | 1.28(0.79-2.09) | 0.32 |
|  |  | A | 166 | 83 |  |  |  |  | 32 | 92 |  |  |
|  | Codominant | GG | 102 | 66 | 1.00 | 0.47 | | | 25 | 53 | 1.00 | 0.55 |
|  |  | AG | 118 | 57 | 0.76 (0.48-1.18) |  | | | 24 | 60 | 1.08 (0.54-2.17) |  |
|  |  | AA | 24 | 13 | 0.90 (0.42-1.91) |  | | | 4 | 16 | 1.92 (0.56-6.52) |  |
|  | Dominant | GG | 102 | 66 | 1.00 | 0.25 | | | 25 | 53 | 1.00 | 0.6 |
|  |  | AG-AA | 142 | 70 | 0.78 (0.51-1.19) |  | | | 28 | 76 | 1.20 (0.61-2.34) |  |
|  | Recessive | GG-AG | 220 | 123 | 1.00 | 0.93 | | | 49 | 113 | 1.00 | 0.29 |
|  |  | AA | 24 | 13 | 1.03 (0.50-2.12) |  | | | 4 | 16 | 1.84 (0.57-5.96) |  |
|  | Overdominant | GG-AA | 126 | 79 | 1.00 | 0.23 | | | 29 | 69 | 1.00 | 0.9 |
|  |  | AG | 118 | 57 | 0.77 (0.50-1.18) |  | | | 24 | 60 | 0.96 (0.49-1.87) |  |
| rs12720270 | Allele | A | 271 | 162 | 1.18(0.87-1.59) | 0.28 | | | 65 | 143 | 0.78(0.49-1.24) | 0.30 |
|  |  | G | 217 | 110 |  |  |  |  | 41 | 115 |  |  |
|  | Codominant | AA | 73 | 48 | 1.00 | 0.62 | | | 18 | 38 | 1.00 | 0.63 |
|  |  | GA | 125 | 66 | 0.83 (0.51-1.33) |  | | | 29 | 67 | 0.95 (0.45-1.98) |  |
|  |  | GG | 46 | 22 | 0.75 (0.62-2.29) |  | | | 6 | 24 | 1.54 (0.52-4.60) |  |
|  | Dominant | AA | 73 | 48 | 1.00 | 0.35 | | | 18 | 38 | 1.00 | 0.9 |
|  |  | GA-GG | 171 | 88 | 0.81 (0.51-1.27) |  | | | 35 | 91 | 1.05 (0.51-2.14) |  |
|  | Recessive | AA-GA | 198 | 114 | 1.00 | 0.55 | | | 47 | 105 | 1.00 | 0.34 |
|  |  | GG | 46 | 22 | 0.84 (0.48-1.48) |  | | | 6 | 24 | 1.59 (0.59-4.29) |  |
|  | Overdominant | AA-GG | 119 | 70 | 1.00 | 0.67 | | | 24 | 62 | 1.00 | 0.58 |
|  |  | GA | 125 | 66 | 0.91 (0.60-1.39) |  | | | 29 | 67 | 0.83 (0.43-1.62) |  |

NOTE:MPA: microscopic polyangiitis; SNP: single nucleotide polymorphism; OR: odds ratio; CI: confidence interval; The *p*-value, OR, and 95% CI were derived from a logistic regression model adjusted for age and ethnicity; * denotes statistical significance (*p*<0.05).
