# Supplementary material for: Association of tyrosine kinase 2 polymorphisms with susceptibility to microscopic polyangiitis in a Guangxi population
Source: PeerJ. 2024 Dec 23;12:e18735. doi: 10.7717/peerj.18735 (PMC11670758; doi:10.7717/peerj.18735)
Supplement: Supplemental Information 4 [file peerj-12-18735-s004.pdf]

SHEsis

Pls cite "Shi YY, He L. SHEsis, a powerful software platform for analyses of linkage disequilibrium, haplotype construction, and genetic association at polymorphism loci. Cell Res. 2005 Feb;15(2):97-8." if you used this platform for research study, thks.

Given you use the haplotype analysis function, pls cite "Li Z, Zhang Z, He Z, Tang W, Li T, Zeng Z, He L, Shi Y. A partition-ligation-combination-subdivision EM algorithm for haplotype inference with multiallelic markers: update of the SHEsis (http://analysis.bio-x.cn). Cell Res. 2009 Apr;19(4):519-23." too, thks.

Any question, pls contact YongYong Shi.

Results

```
297 controls & 265 cases observed
-------------------------------------
-------------------------------------
Single site tests
-------------------------------------
rs2304256(Allele):
                A(freq)         C(freq)
Case:           326(0.615)      204(0.385)
Control:        355(0.598)      239(0.402)
Odds Ratio=1.075863     %95 CI=[0.846462~1.367435]
Chi2 is 0.357216 while df=1
Fisher's p value is 0.550084
Pearson's p value is 0.550078
-------------------------------------
rs2304256(Genotype):
                A/A(freq)       A/C(freq)       C/C(freq)
Case:           98(0.370)       130(0.491)      37(0.140)
Control:        103(0.347)      149(0.502)      45(0.152)
Chi2 is 0.377934 while df=2
Fisher's p value is 0.827820
Pearson's p value is 0.827814
Hardy-Weinberg equilibrium test for case: chi2=0.343973, df=1, Fisher's p is 0.557572, Pearson's p is 0.557567
Hardy-Weinberg equilibrium test for control: chi2=0.552972, df=1, Fisher's p is 0.457141, Pearson's p is 0.457116
-------------------------------------
rs280519(Allele):
                A(freq)         G(freq)
Case:           175(0.330)      355(0.670)
Control:        198(0.333)      396(0.667)
Odds Ratio=0.985915     %95 CI=[0.768792~1.264359]
Chi2 is 0.012492 while df=1
Fisher's p value is 0.911013
Pearson's p value is 0.910988
-------------------------------------
rs280519(Genotype):
                A/A(freq)       A/G(freq)       G/G(freq)
Case:           29(0.109)       117(0.442)      119(0.449)
Control:        28(0.094)       142(0.478)      127(0.428)
Chi2 is 0.871595 while df=2
Fisher's p value is 0.646773
Pearson's p value is 0.646749
Hardy-Weinberg equilibrium test for case: chi2=0.000908, df=1, Fisher's p is 0.975962, Pearson's p is 0.975949
Hardy-Weinberg equilibrium test for control: chi2=1.704545, df=1, Fisher's p is 0.191755, Pearson's p is 0.191675
-------------------------------------
rs12720270(Allele):
                A(freq)         G(freq)
Case:           305(0.575)      225(0.425)
Control:        336(0.566)      258(0.434)
Odds Ratio=1.040873     %95 CI=[0.821546~1.318754]
Chi2 is 0.110108 while df=1
Fisher's p value is 0.740036
Pearson's p value is 0.740030
-------------------------------------
rs12720270(Genotype):
                A/A(freq)       A/G(freq)       G/G(freq)
Case:           86(0.325)       133(0.502)      46(0.174)
Control:        91(0.306)       154(0.519)      52(0.175)
Chi2 is 0.223837 while df=2
Fisher's p value is 0.894119
```

Pearson's p value is 0.894117
Hardy-Weinberg equilibrium test for case: chi2=0.195722, df=1, Fisher's p is 0.658216, Pearson's p is 0.658217
Hardy-Weinberg equilibrium test for control: chi2=0.906039, df=1, Fisher's p is 0.341214, Pearson's p is 0.341158

Linkage Disequilibrium tests

D':	rs280519	rs12720270
rs2304256	0.995	1.000
rs280519	-	0.995

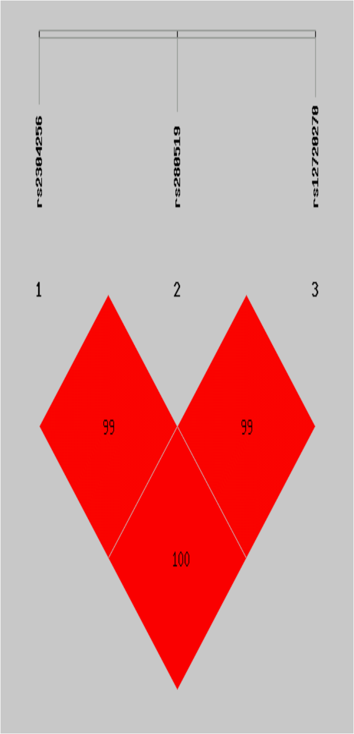

r2:	rs280519	rs12720270
rs2304256	0.756	0.863
rs280519	-	0.652

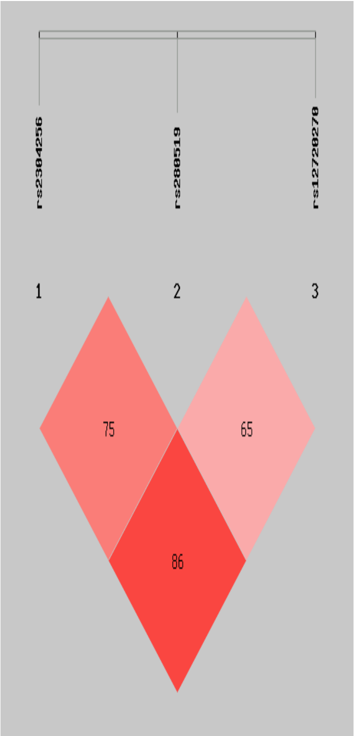

Haplotype analysis
(All those frequency<0.03 will be ignored in analysis.)
Loci chosen for hap-analysis: rs2304256, rs280519, rs12720270

	Case(freq)	Control(freq)	Chi2	MonteCarlo p	Fisher's p	Pearson's p	Odds Ratio [95%CI]
A G A*	303.94(0.573)	336.00(0.566)	0.092	-	0.762052	0.762044	1.037 [0.819~1.314]
A G G*	21.00(0.040)	19.00(0.032)	0.485	-	0.486203	0.486186	1.251 [0.665~2.354]
C A G*	173.94(0.328)	198.00(0.333)	0.025	-	0.873223	0.873198	0.980 [0.764~1.257]
C G G*	30.06(0.057)	41.00(0.069)	0.701	-	0.402397	0.402358	0.813 [0.500~1.321]
A A A	1.06(0.002)	0.00(0.000)	-	-	-	-	-

```
-------------
Global result:
Total control=594.0, total case=530.0
Global chi2 is 1.181075 while df=3 (frequency<0.03 in both control & case has been dropped.)
Fisher's p value is 0.757552
Pearson's p value is 0.757529
-------------------------------------
```

Choose the analysis you need :	☑ Single site analysis	☑ Pair-loci D'/r2 value	☑ Haplotype analysis
Number of sites :	3		
Selected sites for haplotype analysis:	1 1 1	**e.g. "1 1 0 1"** to choose 1st, 2nd & 4th sites for hap-analysis when there is 4 sites in total. Error input will be recognized as default -- **"1 1 1 1 ..." (all chosen).**	
Calculate linkage disequilibrium in:	Both case and control	**You can compare linkage disequilibrium in different groups.**	
Lowest frequency threshold (LFT) for haplotype analysis:	0.03	Default value is 0.03, any number in [0, 1) could be accepted. Haplotype with frequency less than this number will not be considered in analysis.	
Marker names (please use **space** to split variation names) :	rs2304256 rs280519 rs12720270		

Input data of **control**	**Caution:** The format of input data should be -- ID1 G A 1 1 a b ... ID2 A G 1 2 b b ... ID3 G G 1 1 c c ... . Here, the first column refers to the sample ID; the second & third column refer to the	Input data of **case**

A266	C	A	A	G
A267	A	A	G	G
A268	A	A	G	G
A269	C	A	A	G
A270	C	A	A	G
A271	A	A	G	G
A272	C	A	A	G
A273	C	A	A	G
A274	A	A	G	G
A275	C	A	A	G
A276	A	A	G	G
A277	A	A	G	G
A278	C	C	A	G
A279	C	A	A	G
A280	A	A	G	G
A281	C	A	A	G
A282	C	A	A	G
A283	C	C	A	G
A284	C	A	A	G
A285	C	C	A	G
A286	C	A	A	G
A287	C	A	A	G
A288	A	A	G	G
A289	A	A	G	G
A290	C	A	A	G
A291	C	A	A	G
A292	A	A	G	G
A293	C	A	A	G
A294	C	A	A	G

alleles of the 1st site;

fourth & fifth for the 2nd site;

sixth & seventh for the 3rd site;

.....etc.

Pls use **"0"** for the missing alleles.

A1	C	A	A	G
A2	C	C	A	G
A3	A	A	G	G
A4	C	C	A	A
A5	C	C	A	A
A6	C	C	A	A
A7	C	A	A	G
A8	A	A	G	G
A9	C	A	A	G
A10	A	A	G	G
A11	C	A	A	G
A12	C	C	A	A
A13	A	A	G	G
A14	C	A	A	A
A15	C	A	A	G
A16	A	A	G	G
A17	C	A	A	G
A18	C	A	A	G
A19	A	A	G	G
A20	C	A	A	G
A21	A	A	G	G
A22	C	A	A	G
A23	A	A	G	G
A24	C	C	A	A
A25	A	A	G	G
A26	C	C	A	G
A27	C	A	G	G
A28	C	C	A	G
A29	A	A	G	G

Calculate! Clear
