# Supplementary material for: Association of tyrosine kinase 2 polymorphisms with susceptibility to microscopic polyangiitis in a Guangxi population
Source: PeerJ. 2024 Dec 23;12:e18735. doi: 10.7717/peerj.18735 (PMC11670758; doi:10.7717/peerj.18735)
Supplement: Supplemental Information 5 [file peerj-12-18735-s005.pdf]

SNPStats results

Index

Descriptive statistics
Single-SNP analysis
- rs4256
- rs0519
- rs0270

Multiple-SNP analysis
- Linkage disequilibrium analysis
- Haplotype analysis

Descriptive statistics

Response variable: status Type: categorical

	n	missing	unique
**All subjects**	562	0	2
**status=0-control**	297 (52.85%)	---	---
**status=1-cese**	265 (47.15%)	---	---

Covariate: age Type: quantitative

	n	missing	unique	mean	.05	.10	.25	.50	.75	.90	.95
**All subjects**	562	0	67	51.51	26	30	41	52	63	70	75
**status = 0-control**	297	0	62	47.28	25.8	29	38	48	55	64	70
**status = 1-cese**	265	0	63	56.25	26	32.4	47	59	67	74	76.8

lowest: 18, 19, 20, 21, 22 highest: 80, 81, 82, 84, 86

Covariate: ethnicity Type: categorical

	n	missing	unique
**All subjects**	562	0	2
**status=0-control**	297	0	2
**status=1-cese**	265	0	2

	1	2
**All subjects**	383 (68%)	179 (32%)
**status=0-control**	220 (74%)	77 (26%)
**status=1-cese**	163 (62%)	102 (38%)

Covariate: gender Type: categorical

	n	missing	unique
**All subjects**	562	0	2
**status=0-control**	297	0	2
**status=1-cese**	265	0	2

	FeMale	Male
**All subjects**	342 (61%)	220 (39%)
**status=0-control**	173 (58%)	124 (42%)
**status=1-cese**	169 (64%)	96 (36%)

Single-SNP analysis

SNP: rs4256

Percentage of typed samples: 562/562 (100%)

rs4256 allele frequencies (n=562)

	All subjects		status=0-control		status=1-cese	
**Allele**	**Count**	**Proportion**	**Count**	**Proportion**	**Count**	**Proportion**
A	681	0.61	355	0.6	326	0.62
C	443	0.39	239	0.4	204	0.38

rs4256 genotype frequencies (n=562)						
	**All subjects**		**status=0-control**		**status=1-cese**	
**Genotype**	**Count**	**Proportion**	**Count**	**Proportion**	**Count**	**Proportion**
A/A	201	0.36	103	0.35	98	0.37
A/C	279	0.5	149	0.5	130	0.49
C/C	82	0.15	45	0.15	37	0.14

rs4256 exact test for Hardy-Weinberg equilibrium (n=562)						
	**N11**	**N12**	**N22**	**N1**	**N2**	**P-value**
**All subjects**	201	279	82	681	443	0.38
**status=0-control**	103	149	45	355	239	0.55
**status=1-cese**	98	130	37	326	204	0.61

rs4256 association with response status (n=562, adjusted by age+ethnicity+gender)							
**Model**	**Genotype**	**status=0-control**	**status=1-cese**	**OR (95% CI)**	**P-value**	**AIC**	**BIC**
Codominant	A/A	103 (34.7%)	98 (37%)	1.00	0.79	721.2	747.1
	C/A	149 (50.2%)	130 (49.1%)	0.87 (0.60-1.29)			
	C/C	45 (15.2%)	37 (14%)	0.95 (0.55-1.65)			
Dominant	A/A	103 (34.7%)	98 (37%)	1.00	0.54	719.2	740.9
	C/A-C/C	194 (65.3%)	167 (63%)	0.89 (0.62-1.28)			
Recessive	A/A-C/A	252 (84.8%)	228 (86%)	1.00	0.92	719.6	741.3
	C/C	45 (15.2%)	37 (14%)	1.03 (0.62-1.70)			
Overdominant	A/A-C/C	148 (49.8%)	135 (50.9%)	1.00	0.51	719.2	740.8
	C/A	149 (50.2%)	130 (49.1%)	0.89 (0.62-1.26)			
Log-additive	---	---	---	0.95 (0.73-1.23)	0.7	719.5	741.1

Interaction analysis with covariate gender

rs4256 and gender cross-classification interaction table (n=562, adjusted by age+ethnicity)						
	**FeMale**			**Male**		
	**status=0-control**	**status=1-cese**	**OR (95% CI)**	**status=0-control**	**status=1-cese**	**OR (95% CI)**
**A/A**	67	55	1.00	36	43	1.19 (0.65-2.17)
**C/A**	81	92	1.21 (0.74-1.98)	68	38	0.63 (0.36-1.10)
**C/C**	25	22	0.90 (0.44-1.86)	20	15	1.19 (0.53-2.69)
**Interaction p-value:** 0.065						

gender within rs4256 (n=562, adjusted by age+ethnicity)				
		**status=0-control**	**status=1-cese**	**OR (95% CI)**
**A/A**	**FeMale**	67	55	1.00
	**Male**	36	43	1.19 (0.65-2.17)
		**status=0-control**	**status=1-cese**	**OR (95% CI)**
**C/A**	**FeMale**	81	92	1.00
	**Male**	68	38	**0.52 (0.31-0.87)**
		**status=0-control**	**status=1-cese**	**OR (95% CI)**
**C/C**	**FeMale**	25	22	1.00
	**Male**	20	15	1.32 (0.51-3.41)
**Test for interaction in the trend:** 0.57				

rs4256 within gender (n=562, adjusted by age+ethnicity)				
**FeMale**		**status=0-control**	**status=1-cese**	**OR (95% CI)**
	**A/A**	67	55	1.00

	C/A	81	92	1.21 (0.74-1.98)
	C/C	25	22	0.90 (0.44-1.86)
Male		status=0-control	status=1-cese	OR (95% CI)
	A/A	36	43	1.00
	C/A	68	38	0.53 (0.28-0.98)
	C/C	20	15	1.00 (0.42-2.37)
**Test for interaction in the trend:** 0.065				

SNP: rs0519

Percentage of typed samples: 562/562 (100%)

rs0519 allele frequencies (n=562)						
	All subjects		status=0-control		status=1-cese	
**Allele**	**Count**	**Proportion**	**Count**	**Proportion**	**Count**	**Proportion**
G	751	0.67	396	0.67	355	0.67
A	373	0.33	198	0.33	175	0.33

rs0519 genotype frequencies (n=562)						
	All subjects		status=0-control		status=1-cese	
**Genotype**	**Count**	**Proportion**	**Count**	**Proportion**	**Count**	**Proportion**
A/A	57	0.1	28	0.09	29	0.11
G/A	259	0.46	142	0.48	117	0.44
G/G	246	0.44	127	0.43	119	0.45

rs0519 exact test for Hardy-Weinberg equilibrium (n=562)						
	**N11**	**N12**	**N22**	**N1**	**N2**	**P-value**
**All subjects**	246	259	57	751	373	0.39
**status=0-control**	127	142	28	396	198	0.24
**status=1-cese**	119	117	29	355	175	1

rs0519 association with response status (n=562, adjusted by age+ethnicity+gender)

Model	Genotype	status=0-control	status=1-cese	OR (95% CI)	P-value	AIC	BIC
Codominant	G/G	127 (42.8%)	119 (44.9%)	1.00	0.37	719.6	745.6
	A/G	142 (47.8%)	117 (44.1%)	0.82 (0.57-1.20)			
	A/A	28 (9.4%)	29 (10.9%)	1.22 (0.66-2.28)			
Dominant	G/G	127 (42.8%)	119 (44.9%)	1.00	0.5	719.2	740.8
	A/G-A/A	170 (57.2%)	146 (55.1%)	0.88 (0.62-1.26)			
Recessive	G/G-A/G	269 (90.6%)	236 (89.1%)	1.00	0.32	718.7	740.3
	A/A	28 (9.4%)	29 (10.9%)	1.35 (0.74-2.44)			
Overdominant	G/G-A/A	155 (52.2%)	148 (55.9%)	1.00	0.2	718	739.7
	A/G	142 (47.8%)	117 (44.1%)	0.79 (0.56-1.13)			
Log-additive	---	---	---	0.99 (0.76-1.30)	0.94	719.6	741.3

Interaction analysis with covariate gender

rs0519 and gender cross-classification interaction table (n=562, adjusted by age+ethnicity)						
	FeMale			Male		
	status=0-control	status=1-cese	OR (95% CI)	status=0-control	status=1-cese	OR (95% CI)
**G/G**	84	70	1.00	43	49	1.16 (0.67-2.01)
**A/G**	73	81	1.13 (0.70-1.82)	69	36	0.59 (0.34-1.01)
**A/A**	16	18	1.19 (0.54-2.64)	12	11	1.44 (0.56-3.69)
**Interaction p-value:** 0.094						

gender within rs0519 (n=562, adjusted by age+ethnicity)				
**G/G**		status=0-control	status=1-cese	OR (95% CI)
	**FeMale**	84	70	1.00

		status=0-control	status=1-cese	OR (95% CI)
	**Male**	43	49	1.16 (0.67-2.01)
**A/G**		**status=0-control**	**status=1-cese**	**OR (95% CI)**
	**FeMale**	73	81	1.00
	**Male**	69	36	**0.52 (0.30-0.90)**
**A/A**		**status=0-control**	**status=1-cese**	**OR (95% CI)**
	**FeMale**	16	18	1.00
	**Male**	12	11	1.21 (0.39-3.79)
**Test for interaction in the trend:** 0.32				

**rs0519 within gender (n=562, adjusted by age+ethnicity)**				
**FeMale**		**status=0-control**	**status=1-cese**	**OR (95% CI)**
	**G/G**	84	70	1.00
	**A/G**	73	81	1.13 (0.70-1.82)
	**A/A**	16	18	1.19 (0.54-2.64)
**Male**		**status=0-control**	**status=1-cese**	**OR (95% CI)**
	**G/G**	43	49	1.00
	**A/G**	69	36	**0.51 (0.28-0.92)**
	**A/A**	12	11	1.24 (0.47-3.31)
**Test for interaction in the trend:** 0.094				

SNP: rs0270

Percentage of typed samples: 562/562 (100%)

**rs0270 allele frequencies (n=562)**						
	**All subjects**		**status=0-control**		**status=1-cese**	
**Allele**	**Count**	**Proportion**	**Count**	**Proportion**	**Count**	**Proportion**
A	641	0.57	336	0.57	305	0.58
G	483	0.43	258	0.43	225	0.42

**rs0270 genotype frequencies (n=562)**						
	**All subjects**		**status=0-control**		**status=1-cese**	
**Genotype**	**Count**	**Proportion**	**Count**	**Proportion**	**Count**	**Proportion**
A/A	177	0.31	91	0.31	86	0.32
A/G	287	0.51	154	0.52	133	0.5
G/G	98	0.17	52	0.18	46	0.17

**rs0270 exact test for Hardy-Weinberg equilibrium (n=562)**						
	**N11**	**N12**	**N22**	**N1**	**N2**	**P-value**
**All subjects**	177	287	98	641	483	0.34
**status=0-control**	91	154	52	336	258	0.41
**status=1-cese**	86	133	46	305	225	0.71

rs0270 association with response status (n=562, adjusted by age+ethnicity+gender)

**Model**	**Genotype**	**status=0-control**	**status=1-cese**	**OR (95% CI)**	**P-value**	**AIC**	**BIC**
Codominant	A/A	91 (30.6%)	86 (32.5%)	1.00	0.63	720.7	746.7
	G/A	154 (51.9%)	133 (50.2%)	0.83 (0.56-1.24)			
	G/G	52 (17.5%)	46 (17.4%)	0.97 (0.57-1.66)			
Dominant	A/A	91 (30.6%)	86 (32.5%)	1.00	0.46	719.1	740.7
	G/A-G/G	206 (69.4%)	179 (67.5%)	0.87 (0.59-1.26)			
Recessive	A/A-G/A	245 (82.5%)	219 (82.6%)	1.00	0.73	719.5	741.2
	G/G	52 (17.5%)	46 (17.4%)	1.09 (0.68-1.74)			
Overdominant	A/A-G/G	143 (48.1%)	132 (49.8%)	1.00	0.34	718.7	740.4
	G/A	154 (51.9%)	133 (50.2%)	0.84 (0.59-1.20)			
Log-additive	---	---	---	0.96 (0.74-1.24)	0.75	719.5	741.2

Interaction analysis with covariate gender

rs0270 and gender cross-classification interaction table (n=562, adjusted by age+ethnicity)						
	FeMale			Male		
	status=0-control	status=1-cese	OR (95% CI)	status=0-control	status=1-cese	OR (95% CI)
**A/A**	58	49	1.00	33	37	1.03 (0.54-1.95)
**G/A**	85	92	1.06 (0.63-1.78)	69	41	0.59 (0.33-1.05)
**G/G**	30	28	0.83 (0.42-1.66)	22	18	1.26 (0.58-2.75)
**Interaction p-value:** 0.1						

gender within rs0270 (n=562, adjusted by age+ethnicity)				
		status=0-control	status=1-cese	OR (95% CI)
**A/A**	**FeMale**	58	49	1.00
	**Male**	33	37	1.03 (0.54-1.95)
		status=0-control	status=1-cese	OR (95% CI)
**G/A**	**FeMale**	85	92	1.00
	**Male**	69	41	**0.55 (0.33-0.93)**
		status=0-control	status=1-cese	OR (95% CI)
**G/G**	**FeMale**	30	28	1.00
	**Male**	22	18	1.51 (0.63-3.63)
**Test for interaction in the trend:** 0.85				

rs0270 within gender (n=562, adjusted by age+ethnicity)				
		status=0-control	status=1-cese	OR (95% CI)
**FeMale**	**A/A**	58	49	1.00
	**G/A**	85	92	1.06 (0.63-1.78)
	**G/G**	30	28	0.83 (0.42-1.66)
		status=0-control	status=1-cese	OR (95% CI)
**Male**	**A/A**	33	37	1.00
	**G/A**	69	41	0.57 (0.30-1.08)
	**G/G**	22	18	1.22 (0.53-2.82)
**Test for interaction in the trend:** 0.1				

Multiple-SNP analysis

Linkage disequilibrium analysis

D statistic

	rs4256	rs0519	rs0270
rs4256	.	0.2001	0.2247
rs0519	.	.	0.1883
rs0270	.	.	.

D' statistic

	rs4256	rs0519	rs0270
rs4256	.	0.9953	0.9997
rs0519	.	.	0.9948
rs0270	.	.	.

r statistic

	rs4256	rs0519	rs0270
rs4256	.	0.8697	0.9289
rs0519	.	.	0.8076
rs0270	.	.	.

P-values

	rs4256	rs0519	rs0270

rs4256	.	0	0
rs0519	.	.	0
rs0270	.	.	.

Haplotype analysis

Haplotype frequencies estimation (n=562)

	rs4256	rs0519	rs0270	Total	group.0.control	group.1.cese	Cumulative frequency
1	A	G	A	0.5693	0.5657	0.5735	0.5693
2	C	A	G	0.3309	0.3333	0.3282	0.9002
3	C	G	G	0.0632	0.069	0.0567	0.9635
4	A	G	G	0.0356	0.032	0.0396	0.999
5	A	A	A	0.001	NA	0.002	1

Haplotype association with response (n=562, adjusted by age+ethnicity+gender)

	rs4256	rs0519	rs0270	Freq	OR (95% CI)	P-value
1	A	G	A	0.5693	1.00	---
2	C	A	G	0.3309	0.97 (0.73 - 1.28)	0.82
3	C	G	G	0.0632	0.87 (0.50 - 1.50)	0.61
4	A	G	G	0.0356	1.05 (0.54 - 2.05)	0.89

Global haplotype association p-value: 0.96

Haplotype interaction analysis with covariate gender

Haplotype and gender cross-classification interaction table (n=562, adjusted by age+ethnicity)

		FeMale	Male
**Haplotype**	**Frequency**	**OR (95% CI)**	**OR (95% CI)**
**AGA**	0.5693	1.00	0.78 (0.44 - 1.40)
**CAG**	0.3309	1.02 (0.71 - 1.47)	0.69 (0.42 - 1.13)
**AGG**	0.0356	0.63 (0.26 - 1.53)	1.67 (0.58 - 4.84)
**CGG**	0.0632	0.80 (0.40 - 1.58)	0.88 (0.34 - 2.27)

Interaction p-value: 0.25

Haplotypes within gender (n=562, adjusted by age+ethnicity)

		FeMale	Male
**Haplotype**	**Frequency**	**OR (95% CI)**	**OR (95% CI)**
**AGA**	0.5693	1.00	1.00
**CAG**	0.3309	1.02 (0.71 - 1.47)	0.88 (0.56 - 1.39)
**AGG**	0.0356	0.63 (0.26 - 1.53)	2.14 (0.77 - 5.95)
**CGG**	0.0632	0.80 (0.40 - 1.58)	1.12 (0.45 - 2.80)

gender whithin haplotypes (n=562, adjusted by age+ethnicity)

		FeMale	Male
**Haplotype**	**Frequency**	**OR (95% CI)**	**OR (95% CI)**
**AGA**	0.5693	1.00	0.78 (0.44 - 1.40)
**CAG**	0.3309	1.00	0.68 (0.44 - 1.04)
**AGG**	0.0356	1.00	2.64 (0.71 - 9.76)
**CGG**	0.0632	1.00	1.10 (0.37 - 3.26)

<<< Step 3: Customize analysis
