# Supplementary material for: Association of tyrosine kinase 2 polymorphisms with susceptibility to microscopic polyangiitis in a Guangxi population
Source: PeerJ. 2024 Dec 23;12:e18735. doi: 10.7717/peerj.18735 (PMC11670758; doi:10.7717/peerj.18735)
Supplement: Supplemental Information 6 [file peerj-12-18735-s006.pdf]

SNPStats results

Index

Descriptive statistics
Single-SNP analysis
- rs4256
- rs0519
- rs0270

Multiple-SNP analysis
- Linkage disequilibrium analysis
- Haplotype analysis

Descriptive statistics

Response variable: status Type: categorical

	n	missing	unique
**All subjects**	220	0	2
**status=0-control**	124 (56.36%)	---	---
**status=1-cese**	96 (43.64%)	---	---

Covariate: age Type: quantitative

	n	missing	unique	mean	.05	.10	.25	.50	.75	.90	.95
**All subjects**	220	0	60	51.33	25.95	28	41	52	61	70	75
**status = 0-control**	124	0	50	47.35	25	27	36.75	50	57	64.7	69.85
**status = 1-cese**	96	0	40	56.47	27.5	39.5	49.5	58.5	65.25	74	75.25

lowest: 18, 19, 20, 22, 23 highest: 76, 77, 79, 81, 82

Covariate: ethnicity Type: categorical

	n	missing	unique
**All subjects**	220	0	2
**status=0-control**	124	0	2
**status=1-cese**	96	0	2

	1	2
**All subjects**	157 (71%)	63 (29%)
**status=0-control**	93 (75%)	31 (25%)
**status=1-cese**	64 (67%)	32 (33%)

Single-SNP analysis

SNP: rs4256

Percentage of typed samples: 220/220 (100%)

rs4256 allele frequencies (n=220)						
	All subjects		status=0-control		status=1-cese	
**Allele**	**Count**	**Proportion**	**Count**	**Proportion**	**Count**	**Proportion**
A	264	0.6	140	0.56	124	0.65
C	176	0.4	108	0.44	68	0.35

rs4256 genotype frequencies (n=220)						
	All subjects		status=0-control		status=1-cese	
**Genotype**	**Count**	**Proportion**	**Count**	**Proportion**	**Count**	**Proportion**
A/A	79	0.36	36	0.29	43	0.45
A/C	106	0.48	68	0.55	38	0.4
C/C	35	0.16	20	0.16	15	0.16

rs4256 exact test for Hardy-Weinberg equilibrium (n=220)						
	**N11**	**N12**	**N22**	**N1**	**N2**	**P-value**
**All subjects**	79	106	35	264	176	1
**status=0-control**	36	68	20	140	108	0.27
**status=1-cese**	43	38	15	124	68	0.19

rs4256 association with response status (n=220, adjusted by age+ethnicity)							
**Model**	**Genotype**	**status=0-control**	**status=1-cese**	**OR (95% CI)**	**P-value**	**AIC**	**BIC**
Codominant	A/A	36 (29%)	43 (44.8%)	1.00	0.082	279.7	296.7
	C/A	68 (54.8%)	38 (39.6%)	**0.53 (0.28-0.99)**			
	C/C	20 (16.1%)	15 (15.6%)	1.05 (0.43-2.54)			
Dominant	A/A	36 (29%)	43 (44.8%)	1.00	0.11	280.2	293.8
	C/A-C/C	88 (71%)	53 (55.2%)	0.62 (0.34-1.12)			
Recessive	A/A-C/A	104 (83.9%)	81 (84.4%)	1.00	0.31	281.7	295.3
	C/C	20 (16.1%)	15 (15.6%)	1.52 (0.67-3.42)			
Overdominant	A/A-C/C	56 (45.2%)	58 (60.4%)	1.00	0.025	277.7	291.3
	C/A	68 (54.8%)	38 (39.6%)	**0.52 (0.29-0.93)**			
Log-additive	---	---	---	0.87 (0.57-1.34)	0.54	282.4	295.9

Interaction analysis with covariate ethnicity

rs4256 and ethnicity cross-classification interaction table (n=220, adjusted by age)						
	**1**			**2**		
	**status=0-control**	**status=1-cese**	**OR (95% CI)**	**status=0-control**	**status=1-cese**	**OR (95% CI)**
**A/A**	24	27	1.00	12	16	2.01 (0.73-5.54)
**C/A**	52	25	0.50 (0.23-1.08)	16	13	1.18 (0.44-3.14)
**C/C**	17	12	1.07 (0.39-2.93)	3	3	1.86 (0.31-11.24)
**Interaction p-value:** 0.95						

ethnicity within rs4256 (n=220, adjusted by age)				
		**status=0-control**	**status=1-cese**	**OR (95% CI)**
**A/A**	**1**	24	27	1.00
	**2**	12	16	2.01 (0.73-5.54)
		**status=0-control**	**status=1-cese**	**OR (95% CI)**
**C/A**	**1**	52	25	1.00
	**2**	16	13	2.33 (0.92-5.92)
		**status=0-control**	**status=1-cese**	**OR (95% CI)**
**C/C**	**1**	17	12	1.00
	**2**	3	3	1.75 (0.27-11.18)
**Test for interaction in the trend:** 0.79				

rs4256 within ethnicity (n=220, adjusted by age)				
		**status=0-control**	**status=1-cese**	**OR (95% CI)**
**1**	**A/A**	24	27	1.00
	**C/A**	52	25	0.50 (0.23-1.08)
	**C/C**	17	12	1.07 (0.39-2.93)
		**status=0-control**	**status=1-cese**	**OR (95% CI)**
**2**	**A/A**	12	16	1.00
	**C/A**	16	13	0.58 (0.19-1.77)
	**C/C**	3	3	0.92 (0.15-5.88)
**Test for interaction in the trend:** 0.95				

SNP: rs0519

Percentage of typed samples: 220/220 (100%)

rs0519 allele frequencies (n=220)

	All subjects		status=0-control		status=1-cese	
**Allele**	**Count**	**Proportion**	**Count**	**Proportion**	**Count**	**Proportion**
G	289	0.66	155	0.62	134	0.7
A	151	0.34	93	0.38	58	0.3

rs0519 genotype frequencies (n=220)						
	**All subjects**		**status=0-control**		**status=1-cese**	
**Genotype**	**Count**	**Proportion**	**Count**	**Proportion**	**Count**	**Proportion**
A/A	23	0.1	12	0.1	11	0.11
G/A	105	0.48	69	0.56	36	0.38
G/G	92	0.42	43	0.35	49	0.51

rs0519 exact test for Hardy-Weinberg equilibrium (n=220)						
	**N11**	**N12**	**N22**	**N1**	**N2**	**P-value**
**All subjects**	92	105	23	289	151	0.46
**status=0-control**	43	69	12	155	93	0.055
**status=1-cese**	49	36	11	134	58	0.33

rs0519 association with response status (n=220, adjusted by age+ethnicity)							
**Model**	**Genotype**	**status=0-control**	**status=1-cese**	**OR (95% CI)**	**P-value**	**AIC**	**BIC**
Codominant	G/G	43 (34.7%)	49 (51%)	1.00	0.039	278.3	295.2
	A/G	69 (55.6%)	36 (37.5%)	**0.51 (0.28-0.93)**			
	A/A	12 (9.7%)	11 (11.5%)	1.30 (0.47-3.57)			
Dominant	G/G	43 (34.7%)	49 (51%)	1.00	0.078	279.6	293.2
	A/G-A/A	81 (65.3%)	47 (49%)	0.60 (0.33-1.06)			
Recessive	G/G-A/G	112 (90.3%)	85 (88.5%)	1.00	0.2	281.1	294.7
	A/A	12 (9.7%)	11 (11.5%)	1.88 (0.72-4.91)			
Overdominant	G/G-A/A	55 (44.4%)	60 (62.5%)	1.00	0.013	276.5	290.1
	A/G	69 (55.6%)	36 (37.5%)	**0.48 (0.27-0.86)**			
Log-additive	---	---	---	0.84 (0.54-1.31)	0.44	282.1	295.7

Interaction analysis with covariate ethnicity

rs0519 and ethnicity cross-classification interaction table (n=220, adjusted by age)						
	**1**			**2**		
	**status=0-control**	**status=1-cese**	**OR (95% CI)**	**status=0-control**	**status=1-cese**	**OR (95% CI)**
**G/G**	29	31	1.00	14	18	1.92 (0.75-4.91)
**A/G**	53	24	0.48 (0.23-1.00)	16	12	1.10 (0.42-2.90)
**A/A**	11	9	1.20 (0.39-3.63)	1	2	3.61 (0.27-48.16)
**Interaction p-value:** 0.93						

ethnicity within rs0519 (n=220, adjusted by age)				
**G/G**		**status=0-control**	**status=1-cese**	**OR (95% CI)**
	**1**	29	31	1.00
	**2**	14	18	1.92 (0.75-4.91)
**A/G**		**status=0-control**	**status=1-cese**	**OR (95% CI)**
	**1**	53	24	1.00
	**2**	16	12	2.30 (0.89-5.97)
**A/A**		**status=0-control**	**status=1-cese**	**OR (95% CI)**
	**1**	11	9	1.00
	**2**	1	2	3.02 (0.20-44.55)
**Test for interaction in the trend:** 0.94				

rs0519 within ethnicity (n=220, adjusted by age)				
**1**		**status=0-control**	**status=1-cese**	**OR (95% CI)**
	**G/G**	29	31	1.00

	A/G	53	24	0.48 (0.23-1.00)
	A/A	11	9	1.20 (0.39-3.63)
2		**status=0-control**	**status=1-cese**	**OR (95% CI)**
	G/G	14	18	1.00
	A/G	16	12	0.57 (0.19-1.69)
	A/A	1	2	1.88 (0.14-25.84)

Test for interaction in the trend: 0.93

SNP: rs0270

Percentage of typed samples: 220/220 (100%)

rs0270 allele frequencies (n=220)						
	All subjects		status=0-control		status=1-cese	
**Allele**	**Count**	**Proportion**	**Count**	**Proportion**	**Count**	**Proportion**
A	250	0.57	135	0.54	115	0.6
G	190	0.43	113	0.46	77	0.4

rs0270 genotype frequencies (n=220)						
	All subjects		status=0-control		status=1-cese	
**Genotype**	**Count**	**Proportion**	**Count**	**Proportion**	**Count**	**Proportion**
A/A	70	0.32	33	0.27	37	0.39
A/G	110	0.5	69	0.56	41	0.43
G/G	40	0.18	22	0.18	18	0.19

rs0270 exact test for Hardy-Weinberg equilibrium (n=220)						
	**N11**	**N12**	**N22**	**N1**	**N2**	**P-value**
**All subjects**	70	110	40	250	190	0.89
**status=0-control**	33	69	22	135	113	0.21
**status=1-cese**	37	41	18	115	77	0.29

rs0270 association with response status (n=220, adjusted by age+ethnicity)

Model	Genotype	status=0-control	status=1-cese	OR (95% CI)	P-value	AIC	BIC
Codominant	A/A	33 (26.6%)	37 (38.5%)	1.00	0.071	279.4	296.4
	G/A	69 (55.6%)	41 (42.7%)	0.57 (0.30-1.09)			
	G/G	22 (17.7%)	18 (18.8%)	1.32 (0.55-3.14)			
Dominant	A/A	33 (26.6%)	37 (38.5%)	1.00	0.25	281.4	295
	G/A-G/G	91 (73.4%)	59 (61.5%)	0.70 (0.38-1.28)			
Recessive	A/A-G/A	102 (82.3%)	78 (81.2%)	1.00	0.12	280.3	293.9
	G/G	22 (17.7%)	18 (18.8%)	1.85 (0.85-4.03)			
Overdominant	A/A-G/G	55 (44.4%)	55 (57.3%)	1.00	0.027	277.8	291.4
	G/A	69 (55.6%)	41 (42.7%)	**0.52 (0.29-0.93)**			
Log-additive	---	---	---	1.01 (0.66-1.54)	0.97	282.7	296.3

Interaction analysis with covariate ethnicity

rs0270 and ethnicity cross-classification interaction table (n=220, adjusted by age)						
	1			2		
	**status=0-control**	**status=1-cese**	**OR (95% CI)**	**status=0-control**	**status=1-cese**	**OR (95% CI)**
**A/A**	23	24	1.00	10	13	2.49 (0.82-7.60)
**G/A**	51	26	0.59 (0.27-1.30)	18	15	1.31 (0.50-3.42)
**G/G**	19	14	1.36 (0.50-3.70)	3	4	3.00 (0.54-16.61)

Interaction p-value: 0.98

ethnicity within rs0270 (n=220, adjusted by age)				
**A/A**		**status=0-control**	**status=1-cese**	**OR (95% CI)**
	**1**	23	24	1.00

		status=0-control	status=1-cese	OR (95% CI)
	2	10	13	2.49 (0.82-7.60)
G/A		status=0-control	status=1-cese	OR (95% CI)
	1	51	26	1.00
	2	18	15	2.21 (0.91-5.38)
G/G		status=0-control	status=1-cese	OR (95% CI)
	1	19	14	1.00
	2	3	4	2.20 (0.39-12.45)
**Test for interaction in the trend:** 0.69				

rs0270 within ethnicity (n=220, adjusted by age)				
1		status=0-control	status=1-cese	OR (95% CI)
	A/A	23	24	1.00
	G/A	51	26	0.59 (0.27-1.30)
	G/G	19	14	1.36 (0.50-3.70)
2		status=0-control	status=1-cese	OR (95% CI)
	A/A	10	13	1.00
	G/A	18	15	0.53 (0.17-1.66)
	G/G	3	4	1.21 (0.20-7.23)
**Test for interaction in the trend:** 0.98				

Multiple-SNP analysis

Linkage disequilibrium analysis

D statistic

	rs4256	rs0519	rs0270
rs4256	.	0.2059	0.2272
rs0519	.	.	0.1949
rs0270	.	.	.

D' statistic

	rs4256	rs0519	rs0270
rs4256	.	0.9997	0.9997
rs0519	.	.	0.9997
rs0270	.	.	.

r statistic

	rs4256	rs0519	rs0270
rs4256	.	0.8851	0.9363
rs0519	.	.	0.8289
rs0270	.	.	.

P-values

	rs4256	rs0519	rs0270
rs4256	.	0	0
rs0519	.	.	0
rs0270	.	.	.

Haplotype analysis

Haplotype frequencies estimation (n=220)

	rs4256	rs0519	rs0270	Total	group.0.control	group.1.cese	Cumulative frequency
1	A	G	A	0.5682	0.5444	0.599	0.5682
2	C	A	G	0.3432	0.375	0.3021	0.9114
3	C	G	G	0.0568	0.0605	0.0521	0.9682
4	A	G	G	0.0318	0.0202	0.0469	1

Haplotype association with response (n=220, adjusted by age+ethnicity)

	rs4256	rs0519	rs0270	Freq	OR (95% CI)	P-value
1	A	G	A	0.5682	1.00	---
2	C	A	G	0.3432	0.89 (0.56 - 1.42)	0.63
3	C	G	G	0.0568	1.16 (0.46 - 2.94)	0.76
4	A	G	G	0.0318	2.21 (0.78 - 6.22)	0.14
**Global haplotype association p-value:** 0.41						

Haplotype interaction analysis with covariate ethnicity

Haplotype and ethnicity cross-classification interaction table (n=220, adjusted by age)			
		**1**	**2**
**Haplotype**	**Frequency**	**OR (95% CI)**	**OR (95% CI)**
**AGA**	0.5682	1.00	2.42 (0.88 - 6.67)
**CAG**	0.3432	0.92 (0.54 - 1.57)	2.02 (0.80 - 5.07)
**AGG**	0.0318	2.63 (0.76 - 9.16)	3.47 (0.56 - 21.59)
**CGG**	0.0568	1.36 (0.45 - 4.16)	1.94 (0.34 - 10.96)
**Interaction p-value:** 0.92			

Haplotypes within ethnicity (n=220, adjusted by age)			
		**1**	**2**
**Haplotype**	**Frequency**	**OR (95% CI)**	**OR (95% CI)**
**AGA**	0.5682	1.00	1.00
**CAG**	0.3432	0.92 (0.54 - 1.57)	0.84 (0.33 - 2.10)
**AGG**	0.0318	2.63 (0.76 - 9.16)	1.43 (0.22 - 9.29)
**CGG**	0.0568	1.36 (0.45 - 4.16)	0.80 (0.15 - 4.32)

ethnicity whithin haplotypes (n=220, adjusted by age)			
		**1**	**2**
**Haplotype**	**Frequency**	**OR (95% CI)**	**OR (95% CI)**
**AGA**	0.5682	1.00	2.42 (0.88 - 6.67)
**CAG**	0.3432	1.00	2.20 (0.97 - 4.98)
**AGG**	0.0318	1.00	1.32 (0.16 - 11.01)
**CGG**	0.0568	1.00	1.42 (0.21 - 9.83)

<<< Step 3: Customize analysis
