# Supplementary material for: Association of tyrosine kinase 2 polymorphisms with susceptibility to microscopic polyangiitis in a Guangxi population
Source: PeerJ. 2024 Dec 23;12:e18735. doi: 10.7717/peerj.18735 (PMC11670758; doi:10.7717/peerj.18735)
Supplement: Supplemental Information 7 [file peerj-12-18735-s007.pdf]

SNPStats results

Index

Descriptive statistics
Single-SNP analysis
- rs4256
- rs0519
- rs0270

Multiple-SNP analysis
- Linkage disequilibrium analysis
- Haplotype analysis

Descriptive statistics

Response variable: status Type: categorical

	n	missing	unique
**All subjects**	342	0	2
**status=0-control**	173 (50.58%)	---	---
**status=1-cese**	169 (49.42%)	---	---

Covariate: age Type: quantitative

	n	missing	unique	mean	.05	.10	.25	.50	.75	.90	.95
**All subjects**	342	0	63	51.63	26.05	31.1	41	53	63	70.9	75.95
**status = 0-control**	173	0	56	47.23	27	31	38	47	55	63.8	70
**status = 1-cese**	169	0	58	56.12	23.8	32	47	59	67	74.2	77

lowest: 18, 19, 20, 21, 23 highest: 78, 80, 81, 84, 86

Covariate: ethnicity Type: categorical

	n	missing	unique
**All subjects**	342	0	2
**status=0-control**	173	0	2
**status=1-cese**	169	0	2

	1	2
**All subjects**	226 (66%)	116 (34%)
**status=0-control**	127 (73%)	46 (27%)
**status=1-cese**	99 (59%)	70 (41%)

Single-SNP analysis

SNP: rs4256

Percentage of typed samples: 342/342 (100%)

rs4256 allele frequencies (n=342)						
	All subjects		status=0-control		status=1-cese	
**Allele**	**Count**	**Proportion**	**Count**	**Proportion**	**Count**	**Proportion**
A	417	0.61	215	0.62	202	0.6
C	267	0.39	131	0.38	136	0.4

rs4256 genotype frequencies (n=342)						
	All subjects		status=0-control		status=1-cese	
**Genotype**	**Count**	**Proportion**	**Count**	**Proportion**	**Count**	**Proportion**
A/A	122	0.36	67	0.39	55	0.33
A/C	173	0.51	81	0.47	92	0.54
C/C	47	0.14	25	0.14	22	0.13

rs4256 exact test for Hardy-Weinberg equilibrium (n=342)						
	**N11**	**N12**	**N22**	**N1**	**N2**	**P-value**
**All subjects**	122	173	47	417	267	0.31
**status=0-control**	67	81	25	215	131	1
**status=1-cese**	55	92	22	202	136	0.11

rs4256 association with response status (n=342, adjusted by age+ethnicity)							
**Model**	**Genotype**	**status=0-control**	**status=1-cese**	**OR (95% CI)**	**P-value**	**AIC**	**BIC**
Codominant	A/A	67 (38.7%)	55 (32.5%)	1.00	0.6	443.6	462.7
	C/A	81 (46.8%)	92 (54.4%)	1.22 (0.74-1.99)			
	C/C	25 (14.4%)	22 (13%)	0.91 (0.44-1.86)			
Dominant	A/A	67 (38.7%)	55 (32.5%)	1.00	0.58	442.3	457.6
	C/A-C/C	106 (61.3%)	114 (67.5%)	1.14 (0.71-1.83)			
Recessive	A/A-C/A	148 (85.5%)	147 (87%)	1.00	0.53	442.2	457.5
	C/C	25 (14.4%)	22 (13%)	0.81 (0.42-1.56)			
Overdominant	A/A-C/C	92 (53.2%)	77 (45.6%)	1.00	0.33	441.6	457
	C/A	81 (46.8%)	92 (54.4%)	1.25 (0.80-1.96)			
Log-additive	---	---	---	1.01 (0.72-1.42)	0.94	442.6	457.9

Interaction analysis with covariate ethnicity

rs4256 and ethnicity cross-classification interaction table (n=342, adjusted by age)						
	**1**			**2**		
	**status=0-control**	**status=1-cese**	**OR (95% CI)**	**status=0-control**	**status=1-cese**	**OR (95% CI)**
**A/A**	51	33	1.00	16	22	**2.52 (1.11-5.72)**
**C/A**	62	53	1.25 (0.69-2.27)	19	39	**2.90 (1.39-6.03)**
**C/C**	14	13	1.38 (0.55-3.46)	11	9	1.25 (0.45-3.50)
**Interaction p-value:** 0.35						

ethnicity within rs4256 (n=342, adjusted by age)				
		**status=0-control**	**status=1-cese**	**OR (95% CI)**
**A/A**	**1**	51	33	1.00
	**2**	16	22	**2.52 (1.11-5.72)**
		**status=0-control**	**status=1-cese**	**OR (95% CI)**
**C/A**	**1**	62	53	1.00
	**2**	19	39	**2.32 (1.17-4.62)**
		**status=0-control**	**status=1-cese**	**OR (95% CI)**
**C/C**	**1**	14	13	1.00
	**2**	11	9	0.91 (0.27-3.07)
**Test for interaction in the trend:** 0.2				

rs4256 within ethnicity (n=342, adjusted by age)				
		**status=0-control**	**status=1-cese**	**OR (95% CI)**
**1**	**A/A**	51	33	1.00
	**C/A**	62	53	1.25 (0.69-2.27)
	**C/C**	14	13	1.38 (0.55-3.46)
		**status=0-control**	**status=1-cese**	**OR (95% CI)**
**2**	**A/A**	16	22	1.00
	**C/A**	19	39	1.15 (0.47-2.80)
	**C/C**	11	9	0.50 (0.16-1.56)
**Test for interaction in the trend:** 0.35				

SNP: rs0519

Percentage of typed samples: 342/342 (100%)

rs0519 allele frequencies (n=342)

	All subjects		status=0-control		status=1-cese	
**Allele**	**Count**	**Proportion**	**Count**	**Proportion**	**Count**	**Proportion**
G	462	0.68	241	0.7	221	0.65
A	222	0.32	105	0.3	117	0.35

rs0519 genotype frequencies (n=342)						
	**All subjects**		**status=0-control**		**status=1-cese**	
**Genotype**	**Count**	**Proportion**	**Count**	**Proportion**	**Count**	**Proportion**
A/A	34	0.1	16	0.09	18	0.11
G/A	154	0.45	73	0.42	81	0.48
G/G	154	0.45	84	0.49	70	0.41

rs0519 exact test for Hardy-Weinberg equilibrium (n=342)						
	**N11**	**N12**	**N22**	**N1**	**N2**	**P-value**
**All subjects**	154	154	34	462	222	0.71
**status=0-control**	84	73	16	241	105	1
**status=1-cese**	70	81	18	221	117	0.5

rs0519 association with response status (n=342, adjusted by age+ethnicity)							
**Model**	**Genotype**	**status=0-control**	**status=1-cese**	**OR (95% CI)**	**P-value**	**AIC**	**BIC**
Codominant	G/G	84 (48.5%)	70 (41.4%)	1.00	0.83	444.2	463.4
	A/G	73 (42.2%)	81 (47.9%)	1.14 (0.71-1.83)			
	A/A	16 (9.2%)	18 (10.7%)	1.20 (0.54-2.65)			
Dominant	G/G	84 (48.5%)	70 (41.4%)	1.00	0.56	442.2	457.6
	A/G-A/A	89 (51.5%)	99 (58.6%)	1.15 (0.73-1.81)			
Recessive	G/G-A/G	157 (90.8%)	151 (89.3%)	1.00	0.76	442.5	457.8
	A/A	16 (9.2%)	18 (10.7%)	1.12 (0.53-2.40)			
Overdominant	G/G-A/A	100 (57.8%)	88 (52.1%)	1.00	0.68	442.4	457.7
	A/G	73 (42.2%)	81 (47.9%)	1.10 (0.70-1.73)			
Log-additive	---	---	---	1.11 (0.78-1.57)	0.56	442.2	457.6

Interaction analysis with covariate ethnicity

rs0519 and ethnicity cross-classification interaction table (n=342, adjusted by age)						
	**1**			**2**		
	**status=0-control**	**status=1-cese**	**OR (95% CI)**	**status=0-control**	**status=1-cese**	**OR (95% CI)**
**G/G**	64	43	1.00	20	27	**2.36 (1.13-4.92)**
**A/G**	52	44	1.19 (0.66-2.12)	21	37	**2.41 (1.21-4.80)**
**A/A**	11	12	1.55 (0.60-4.03)	5	6	1.60 (0.44-5.91)
**Interaction p-value:** 0.63						

ethnicity within rs0519 (n=342, adjusted by age)				
**G/G**		**status=0-control**	**status=1-cese**	**OR (95% CI)**
	**1**	64	43	1.00
	**2**	20	27	**2.36 (1.13-4.92)**
**A/G**		**status=0-control**	**status=1-cese**	**OR (95% CI)**
	**1**	52	44	1.00
	**2**	21	37	**2.03 (1.01-4.08)**
**A/A**		**status=0-control**	**status=1-cese**	**OR (95% CI)**
	**1**	11	12	1.00
	**2**	5	6	1.03 (0.23-4.68)
**Test for interaction in the trend:** 0.41				

rs0519 within ethnicity (n=342, adjusted by age)				
**1**		**status=0-control**	**status=1-cese**	**OR (95% CI)**
	**G/G**	64	43	1.00

		status=0-control	status=1-cese	OR (95% CI)
	A/G	52	44	1.19 (0.66-2.12)
	A/A	11	12	1.55 (0.60-4.03)
2		status=0-control	status=1-cese	OR (95% CI)
	G/G	20	27	1.00
	A/G	21	37	1.02 (0.45-2.35)
	A/A	5	6	0.68 (0.17-2.72)
**Test for interaction in the trend:** 0.63				

SNP: rs0270

Percentage of typed samples: 342/342 (100%)

rs0270 allele frequencies (n=342)						
	All subjects		status=0-control		status=1-cese	
Allele	Count	Proportion	Count	Proportion	Count	Proportion
A	391	0.57	201	0.58	190	0.56
G	293	0.43	145	0.42	148	0.44

rs0270 genotype frequencies (n=342)						
	All subjects		status=0-control		status=1-cese	
Genotype	Count	Proportion	Count	Proportion	Count	Proportion
A/A	107	0.31	58	0.34	49	0.29
A/G	177	0.52	85	0.49	92	0.54
G/G	58	0.17	30	0.17	28	0.17

rs0270 exact test for Hardy-Weinberg equilibrium (n=342)						
	N11	N12	N22	N1	N2	P-value
**All subjects**	107	177	58	391	293	0.32
**status=0-control**	58	85	30	201	145	1
**status=1-cese**	49	92	28	190	148	0.21

rs0270 association with response status (n=342, adjusted by age+ethnicity)

Model	Genotype	status=0-control	status=1-cese	OR (95% CI)	P-value	AIC	BIC
Codominant	A/A	58 (33.5%)	49 (29%)	1.00	0.76	444	463.2
	G/A	85 (49.1%)	92 (54.4%)	1.08 (0.64-1.79)			
	G/G	30 (17.3%)	28 (16.6%)	0.85 (0.43-1.69)			
Dominant	A/A	58 (33.5%)	49 (29%)	1.00	0.95	442.6	457.9
	G/A-G/G	115 (66.5%)	120 (71%)	1.02 (0.62-1.65)			
Recessive	A/A-G/A	143 (82.7%)	141 (83.4%)	1.00	0.5	442.1	457.4
	G/G	30 (17.3%)	28 (16.6%)	0.81 (0.44-1.49)			
Overdominant	A/A-G/G	88 (50.9%)	77 (45.6%)	1.00	0.57	442.2	457.6
	G/A	85 (49.1%)	92 (54.4%)	1.14 (0.72-1.79)			
Log-additive	---	---	---	0.94 (0.68-1.32)	0.74	442.5	457.8

Interaction analysis with covariate ethnicity

rs0270 and ethnicity cross-classification interaction table (n=342, adjusted by age)						
	1			2		
	status=0-control	status=1-cese	OR (95% CI)	status=0-control	status=1-cese	OR (95% CI)
**A/A**	45	29	1.00	13	20	**3.01 (1.24-7.32)**
**G/A**	64	56	1.24 (0.67-2.30)	21	36	**2.32 (1.10-4.89)**
**G/G**	18	14	1.09 (0.45-2.62)	12	14	1.67 (0.65-4.31)
**Interaction p-value:** 0.59						

ethnicity within rs0270 (n=342, adjusted by age)				
**A/A**		status=0-control	status=1-cese	OR (95% CI)
	1	45	29	1.00

		status=0-control	status=1-cese	OR (95% CI)
	2	13	20	3.01 (1.24-7.32)
G/A		status=0-control	status=1-cese	OR (95% CI)
	1	64	56	1.00
	2	21	36	1.87 (0.95-3.67)
G/G		status=0-control	status=1-cese	OR (95% CI)
	1	18	14	1.00
	2	12	14	1.54 (0.51-4.59)
**Test for interaction in the trend:** 0.29				

rs0270 within ethnicity (n=342, adjusted by age)				
1		status=0-control	status=1-cese	OR (95% CI)
	A/A	45	29	1.00
	G/A	64	56	1.24 (0.67-2.30)
	G/G	18	14	1.09 (0.45-2.62)
2		status=0-control	status=1-cese	OR (95% CI)
	A/A	13	20	1.00
	G/A	21	36	0.77 (0.30-1.96)
	G/G	12	14	0.55 (0.18-1.67)
**Test for interaction in the trend:** 0.59				

Multiple-SNP analysis

Linkage disequilibrium analysis

D statistic

	rs4256	rs0519	rs0270
rs4256	.	0.1963	0.2231
rs0519	.	.	0.1839
rs0270	.	.	.

D' statistic

	rs4256	rs0519	rs0270
rs4256	.	0.992	0.9997
rs0519	.	.	0.9912
rs0270	.	.	.

r statistic

	rs4256	rs0519	rs0270
rs4256	.	0.8593	0.9241
rs0519	.	.	0.7937
rs0270	.	.	.

P-values

	rs4256	rs0519	rs0270
rs4256	.	0	0
rs0519	.	.	0
rs0270	.	.	.

Haplotype analysis

Haplotype frequencies estimation (n=342)

	rs4256	rs0519	rs0270	Total	group.0.control	group.1.cese	Cumulative frequency
1	A	G	A	0.5701	0.5809	0.559	0.5701
2	C	A	G	0.323	0.3035	0.343	0.8931
3	C	G	G	0.0674	0.0751	0.0594	0.9604
4	A	G	G	0.038	0.0405	0.0355	0.9984
5	A	A	A	0.0016	NA	0.0032	1

6	A	A	G	0	NA	0	1

Haplotype association with response (n=342, adjusted by age+ethnicity)						
	**rs4256**	**rs0519**	**rs0270**	**Freq**	**OR (95% CI)**	**P-value**
1	A	G	A	0.5701	1.00	---
2	C	A	G	0.323	1.02 (0.71 - 1.46)	0.92
3	C	G	G	0.0674	0.80 (0.40 - 1.58)	0.52
4	A	G	G	0.038	0.65 (0.27 - 1.56)	0.33
rare	*	*	*	0.0016	**724588951.01 (724588950.88 - 724588951.13)**	<0.0001
**Global haplotype association p-value:** 0.43						

Haplotype interaction analysis with covariate ethnicity

Haplotype and ethnicity cross-classification interaction table (n=342, adjusted by age)			
		**1**	**2**
**Haplotype**	**Frequency**	**OR (95% CI)**	**OR (95% CI)**
**AGA**	0.5701	1.00	**2.84 (1.28 - 6.28)**
**CAG**	0.323	1.15 (0.74 - 1.79)	**2.37 (1.24 - 4.52)**
**CGG**	0.0674	1.11 (0.47 - 2.63)	1.41 (0.44 - 4.48)
**AGG**	0.038	0.55 (0.17 - 1.78)	2.30 (0.55 - 9.63)
**rare**	0.0016	Inf	Inf
**Interaction p-value:** 0.74			

Haplotypes within ethnicity (n=342, adjusted by age)			
		**1**	**2**
**Haplotype**	**Frequency**	**OR (95% CI)**	**OR (95% CI)**
**AGA**	0.5701	1.00	1.00
**CAG**	0.323	1.15 (0.74 - 1.79)	0.83 (0.44 - 1.57)
**CGG**	0.0674	1.11 (0.47 - 2.63)	0.50 (0.17 - 1.48)
**AGG**	0.038	0.55 (0.17 - 1.78)	0.81 (0.20 - 3.34)
**rare**	0.0016	Inf	Inf

ethnicity whithin haplotypes (n=342, adjusted by age)			
		**1**	**2**
**Haplotype**	**Frequency**	**OR (95% CI)**	**OR (95% CI)**
**AGA**	0.5701	1.00	**2.84 (1.28 - 6.28)**
**CAG**	0.323	1.00	**2.06 (1.16 - 3.67)**
**CGG**	0.0674	1.00	1.28 (0.34 - 4.80)
**AGG**	0.038	1.00	4.14 (0.71 - 24.21)
**rare**	0.0016	1.00	0.00 (-Inf - Inf)

<<< Step 3: Customize analysis
