# Supplementary material for: Association of tyrosine kinase 2 polymorphisms with susceptibility to microscopic polyangiitis in a Guangxi population
Source: PeerJ. 2024 Dec 23;12:e18735. doi: 10.7717/peerj.18735 (PMC11670758; doi:10.7717/peerj.18735)
Supplement: Supplemental Information 8 [file peerj-12-18735-s008.pdf]

# SNPStats results

## Index

**Descriptive statistics**
**Single-SNP analysis**
rs4256
rs0519
rs0270
**Multiple-SNP analysis**
Linkage disequilibrium analysis
Haplotype analysis

## Descriptive statistics

### Response variable: status Type: categorical

| | n | missing | unique |
|---|---|---|---|
| **All subjects** | 383 | 0 | 2 |
| **status=0-control** | 220 (57.44%) | --- | --- |
| **status=1-cese** | 163 (42.56%) | --- | --- |

---

### Covariate: age Type: quantitative

| | n | missing | unique | mean | .05 | .10 | .25 | .50 | .75 | .90 | .95 |
|---|---|---|---|---|---|---|---|---|---|---|---|
| **All subjects** | 383 | 0 | 64 | 52.08 | 26 | 30.2 | 41 | 53 | 63 | 70.8 | 75 |
| **status = 0-control** | 220 | 0 | 59 | 48.15 | 26 | 29 | 38 | 48 | 57.25 | 68 | 71.05 |
| **status = 1-cese** | 163 | 0 | 52 | 57.39 | 28.1 | 38.2 | 50.5 | 60 | 67 | 74 | 75.9 |

lowest: 18, 19, 20, 22, 23 highest: 79, 80, 81, 82, 84

---

### Covariate: gender Type: categorical

| | n | missing | unique |
|---|---|---|---|
| **All subjects** | 383 | 0 | 2 |
| **status=0-control** | 220 | 0 | 2 |
| **status=1-cese** | 163 | 0 | 2 |

| | FeMale | Male |
|---|---|---|
| **All subjects** | 226 (59%) | 157 (41%) |
| **status=0-control** | 127 (58%) | 93 (42%) |
| **status=1-cese** | 99 (61%) | 64 (39%) |

---

## Single-SNP analysis

### SNP: rs4256

**Percentage of typed samples:** 383/383 (100%)

| rs4256 allele frequencies (n=383) | | | | | | |
|---|---|---|---|---|---|---|
| | All subjects | | status=0-control | | status=1-cese | |
| **Allele** | **Count** | **Proportion** | **Count** | **Proportion** | **Count** | **Proportion** |
| A | 462 | 0.6 | 264 | 0.6 | 198 | 0.61 |
| C | 304 | 0.4 | 176 | 0.4 | 128 | 0.39 |

| rs4256 genotype frequencies (n=383) | | | | | | |
|---|---|---|---|---|---|---|
| | All subjects | | status=0-control | | status=1-cese | |
| **Genotype** | **Count** | **Proportion** | **Count** | **Proportion** | **Count** | **Proportion** |
| A/A | 135 | 0.35 | 75 | 0.34 | 60 | 0.37 |
| A/C | 192 | 0.5 | 114 | 0.52 | 78 | 0.48 |
| C/C | 56 | 0.15 | 31 | 0.14 | 25 | 0.15 |

| rs4256 exact test for Hardy-Weinberg equilibrium (n=383) | | | | | | |
|---|---|---|---|---|---|---|
| | N11 | N12 | N22 | N1 | N2 | P-value |
| All subjects | 135 | 192 | 56 | 462 | 304 | 0.39 |
| status=0-control | 75 | 114 | 31 | 264 | 176 | 0.26 |
| status=1-cese | 60 | 78 | 25 | 198 | 128 | 1 |

| rs4256 association with response status (n=383, adjusted by age+gender) | | | | | | | |
|---|---|---|---|---|---|---|---|
| Model | Genotype | status=0-control | status=1-cese | OR (95% CI) | P-value | AIC | BIC |
| Codominant | A/A | 75 (34.1%) | 60 (36.8%) | 1.00 | 0.53 | 492.6 | 512.3 |
| | C/A | 114 (51.8%) | 78 (47.9%) | 0.87 (0.55-1.39) | | | |
| | C/C | 31 (14.1%) | 25 (15.3%) | 1.25 (0.64-2.46) | | | |
| Dominant | A/A | 75 (34.1%) | 60 (36.8%) | 1.00 | 0.8 | 491.7 | 507.5 |
| | C/A-C/C | 145 (65.9%) | 103 (63.2%) | 0.94 (0.60-1.47) | | | |
| Recessive | A/A-C/A | 189 (85.9%) | 138 (84.7%) | 1.00 | 0.33 | 490.9 | 506.7 |
| | C/C | 31 (14.1%) | 25 (15.3%) | 1.36 (0.73-2.52) | | | |
| Overdominant | A/A-C/C | 106 (48.2%) | 85 (52.1%) | 1.00 | 0.36 | 491 | 506.8 |
| | C/A | 114 (51.8%) | 78 (47.9%) | 0.82 (0.54-1.26) | | | |
| Log-additive | --- | --- | --- | 1.05 (0.76-1.45) | 0.75 | 491.7 | 507.5 |

---

## SNP: rs0519

**Percentage of typed samples:** 383/383 (100%)

| rs0519 allele frequencies (n=383) | | | | | | |
|---|---|---|---|---|---|---|
| | All subjects | | status=0-control | | status=1-cese | |
| Allele | Count | Proportion | Count | Proportion | Count | Proportion |
| G | 507 | 0.66 | 291 | 0.66 | 216 | 0.66 |
| A | 259 | 0.34 | 149 | 0.34 | 110 | 0.34 |

| rs0519 genotype frequencies (n=383) | | | | | | |
|---|---|---|---|---|---|---|
| | All subjects | | status=0-control | | status=1-cese | |
| Genotype | Count | Proportion | Count | Proportion | Count | Proportion |
| A/A | 43 | 0.11 | 22 | 0.1 | 21 | 0.13 |
| G/A | 173 | 0.45 | 105 | 0.48 | 68 | 0.42 |
| G/G | 167 | 0.44 | 93 | 0.42 | 74 | 0.45 |

| rs0519 exact test for Hardy-Weinberg equilibrium (n=383) | | | | | | |
|---|---|---|---|---|---|---|
| | N11 | N12 | N22 | N1 | N2 | P-value |
| All subjects | 167 | 173 | 43 | 507 | 259 | 0.91 |
| status=0-control | 93 | 105 | 22 | 291 | 149 | 0.37 |
| status=1-cese | 74 | 68 | 21 | 216 | 110 | 0.38 |

| rs0519 association with response status (n=383, adjusted by age+gender) | | | | | | | |
|---|---|---|---|---|---|---|---|
| Model | Genotype | status=0-control | status=1-cese | OR (95% CI) | P-value | AIC | BIC |
| Codominant | G/G | 93 (42.3%) | 74 (45.4%) | 1.00 | 0.32 | 491.5 | 511.3 |
| | A/G | 105 (47.7%) | 68 (41.7%) | 0.82 (0.52-1.30) | | | |
| | A/A | 22 (10%) | 21 (12.9%) | 1.41 (0.69-2.90) | | | |
| Dominant | G/G | 93 (42.3%) | 74 (45.4%) | 1.00 | 0.69 | 491.7 | 507.4 |
| | A/G-A/A | 127 (57.7%) | 89 (54.6%) | 0.92 (0.60-1.41) | | | |
| Recessive | G/G-A/G | 198 (90%) | 142 (87.1%) | 1.00 | 0.21 | 490.2 | 506 |
| | A/A | 22 (10%) | 21 (12.9%) | 1.55 (0.78-3.08) | | | |
| Overdominant | G/G-A/A | 115 (52.3%) | 95 (58.3%) | 1.00 | 0.23 | 490.4 | 506.2 |
| | A/G | 105 (47.7%) | 68 (41.7%) | 0.77 (0.50-1.18) | | | |
| Log-additive | --- | --- | --- | 1.05 (0.76-1.45) | 0.77 | 491.7 | 507.5 |

---

## SNP: rs0270

**Percentage of typed samples:** 383/383 (100%)

| rs0270 allele frequencies (n=383) | | | | | | |
|---|---|---|---|---|---|---|
| | All subjects | | status=0-control | | status=1-cese | |
| Allele | Count | Proportion | Count | Proportion | Count | Proportion |
| A | 439 | 0.57 | 251 | 0.57 | 188 | 0.58 |
| G | 327 | 0.43 | 189 | 0.43 | 138 | 0.42 |

| rs0270 genotype frequencies (n=383) | | | | | | |
|---|---|---|---|---|---|---|
| | All subjects | | status=0-control | | status=1-cese | |
| Genotype | Count | Proportion | Count | Proportion | Count | Proportion |
| A/A | 121 | 0.32 | 68 | 0.31 | 53 | 0.33 |
| A/G | 197 | 0.51 | 115 | 0.52 | 82 | 0.5 |
| G/G | 65 | 0.17 | 37 | 0.17 | 28 | 0.17 |

| rs0270 exact test for Hardy-Weinberg equilibrium (n=383) | | | | | | |
|---|---|---|---|---|---|---|
| | N11 | N12 | N22 | N1 | N2 | P-value |
| **All subjects** | 121 | 197 | 65 | 439 | 327 | 0.35 |
| **status=0-control** | 68 | 115 | 37 | 251 | 189 | 0.41 |
| **status=1-cese** | 53 | 82 | 28 | 188 | 138 | 0.75 |

| rs0270 association with response status (n=383, adjusted by age+gender) | | | | | | | |
|---|---|---|---|---|---|---|---|
| Model | Genotype | status=0-control | status=1-cese | OR (95% CI) | P-value | AIC | BIC |
| Codominant | A/A | 68 (30.9%) | 53 (32.5%) | 1.00 | 0.7 | 493.1 | 512.8 |
| | G/A | 115 (52.3%) | 82 (50.3%) | 0.92 (0.57-1.49) | | | |
| | G/G | 37 (16.8%) | 28 (17.2%) | 1.20 (0.62-2.29) | | | |
| Dominant | A/A | 68 (30.9%) | 53 (32.5%) | 1.00 | 0.94 | 491.8 | 507.6 |
| | G/A-G/G | 152 (69.1%) | 110 (67.5%) | 0.98 (0.62-1.55) | | | |
| Recessive | A/A-G/A | 183 (83.2%) | 135 (82.8%) | 1.00 | 0.44 | 491.2 | 507 |
| | G/G | 37 (16.8%) | 28 (17.2%) | 1.26 (0.70-2.24) | | | |
| Overdominant | A/A-G/G | 105 (47.7%) | 81 (49.7%) | 1.00 | 0.52 | 491.4 | 507.2 |
| | G/A | 115 (52.3%) | 82 (50.3%) | 0.87 (0.57-1.33) | | | |
| Log-additive | --- | --- | --- | 1.06 (0.77-1.46) | 0.72 | 491.7 | 507.5 |

---

## Multiple-SNP analysis

### Linkage disequilibrium analysis

### D statistic

| | rs4256 | rs0519 | rs0270 |
|---|---|---|---|
| rs4256 | . | 0.2025 | 0.2274 |
| rs0519 | . | . | 0.1923 |
| rs0270 | . | . | . |

### D' statistic

| | rs4256 | rs0519 | rs0270 |
|---|---|---|---|
| rs4256 | . | 0.9931 | 0.9997 |
| rs0519 | . | . | 0.9925 |
| rs0270 | . | . | . |

### r statistic

| | rs4256 | rs0519 | rs0270 |
|---|---|---|---|
| rs4256 | . | 0.8751 | 0.9396 |
| rs0519 | . | . | 0.822 |
| rs0270 | . | . | . |

### P-values

| | rs4256 | rs0519 | rs0270 |
|---|---|---|---|
| rs4256 | . | 0 | 0 |
| rs0519 | . | . | 0 |
| rs0270 | . | . | . |

## Haplotype analysis

| Haplotype frequencies estimation (n=383) | | | | | | | |
|---|---|---|---|---|---|---|---|
| | **rs4256** | **rs0519** | **rs0270** | **Total** | **group.0.control** | **group.1.cese** | **Cumulative frequency** |
| 1 | A | G | A | 0.5717 | 0.5705 | 0.5734 | 0.5717 |
| 2 | C | A | G | 0.3367 | 0.3386 | 0.3342 | 0.9084 |
| 3 | C | G | G | 0.0601 | 0.0614 | 0.0585 | 0.9686 |
| 4 | A | G | G | 0.03 | 0.0295 | 0.0307 | 0.9986 |
| 5 | A | A | A | 0.0014 | NA | 0.0033 | 1 |

| Haplotype association with response (n=383, adjusted by age+gender) | | | | | | |
|---|---|---|---|---|---|---|
| | **rs4256** | **rs0519** | **rs0270** | **Freq** | **OR (95% CI)** | **P-value** |
| 1 | A | G | A | 0.5717 | 1.00 | --- |
| 2 | C | A | G | 0.3367 | 1.04 (0.74 - 1.46) | 0.81 |
| 3 | C | G | G | 0.0601 | 1.13 (0.57 - 2.24) | 0.72 |
| 4 | A | G | G | 0.03 | 1.09 (0.47 - 2.55) | 0.84 |
| rare | * | * | * | 0.0014 | **375936981.90 (375936981.75 - 375936982.04)** | <0.0001 |
| **Global haplotype association p-value:** 0.65 | | | | | | |

<<< Step 3: Customize analysis
