# Supplementary material for: Association of tyrosine kinase 2 polymorphisms with susceptibility to microscopic polyangiitis in a Guangxi population
Source: PeerJ. 2024 Dec 23;12:e18735. doi: 10.7717/peerj.18735 (PMC11670758; doi:10.7717/peerj.18735)
Supplement: Supplemental Information 9 [file peerj-12-18735-s009.pdf]

# SNPStats results

## Index

**Descriptive statistics**
**Single-SNP analysis**
- rs4256
- rs0519
- rs0270

**Multiple-SNP analysis**
- Linkage disequilibrium analysis
- Haplotype analysis

## Descriptive statistics

### Response variable: status Type: categorical

| | n | missing | unique |
|---|---|---|---|
| **All subjects** | 179 | 0 | 2 |
| **status=0-control** | 77 (43.02%) | --- | --- |
| **status=1-cese** | 102 (56.98%) | --- | --- |

---

### Covariate: age Type: quantitative

| | n | missing | unique | mean | .05 | .10 | .25 | .50 | .75 | .90 | .95 |
|---|---|---|---|---|---|---|---|---|---|---|---|
| **All subjects** | 179 | 0 | 59 | 50.29 | 22.9 | 29 | 40 | 51 | 61 | 69 | 76.1 |
| **status = 0-control** | 77 | 0 | 41 | 44.81 | 21.8 | 28.6 | 36 | 48 | 52 | 57.4 | 61 |
| **status = 1-cese** | 102 | 0 | 49 | 54.43 | 25.05 | 30 | 42.25 | 58 | 66 | 74 | 77 |

lowest: 18, 19, 20, 21, 22 highest: 77, 78, 80, 82, 86

---

### Covariate: gender Type: categorical

| | n | missing | unique |
|---|---|---|---|
| **All subjects** | 179 | 0 | 2 |
| **status=0-control** | 77 | 0 | 2 |
| **status=1-cese** | 102 | 0 | 2 |

| | FeMale | Male |
|---|---|---|
| **All subjects** | 116 (65%) | 63 (35%) |
| **status=0-control** | 46 (60%) | 31 (40%) |
| **status=1-cese** | 70 (69%) | 32 (31%) |

---

## Single-SNP analysis

### SNP: rs4256

**Percentage of typed samples:** 179/179 (100%)

| rs4256 allele frequencies (n=179) | | | | | | |
|---|---|---|---|---|---|---|
| | All subjects | | status=0-control | | status=1-cese | |
| **Allele** | **Count** | **Proportion** | **Count** | **Proportion** | **Count** | **Proportion** |
| A | 219 | 0.61 | 91 | 0.59 | 128 | 0.63 |
| C | 139 | 0.39 | 63 | 0.41 | 76 | 0.37 |

| rs4256 genotype frequencies (n=179) | | | | | | |
|---|---|---|---|---|---|---|
| | All subjects | | status=0-control | | status=1-cese | |
| **Genotype** | **Count** | **Proportion** | **Count** | **Proportion** | **Count** | **Proportion** |
| A/A | 66 | 0.37 | 28 | 0.36 | 38 | 0.37 |
| A/C | 87 | 0.49 | 35 | 0.45 | 52 | 0.51 |
| C/C | 26 | 0.15 | 14 | 0.18 | 12 | 0.12 |

| rs4256 exact test for Hardy-Weinberg equilibrium (n=179) | | | | | | |
|---|---|---|---|---|---|---|
| | **N11** | **N12** | **N22** | **N1** | **N2** | **P-value** |
| **All subjects** | 66 | 87 | 26 | 219 | 139 | 0.87 |
| **status=0-control** | 28 | 35 | 14 | 91 | 63 | 0.64 |
| **status=1-cese** | 38 | 52 | 12 | 128 | 76 | 0.41 |

| rs4256 association with response status (n=179, adjusted by age+gender) | | | | | | | |
|---|---|---|---|---|---|---|---|
| **Model** | **Genotype** | **status=0-control** | **status=1-cese** | **OR (95% CI)** | **P-value** | **AIC** | **BIC** |
| Codominant | A/A | 28 (36.4%) | 38 (37.2%) | 1.00 | 0.45 | 234.2 | 250.1 |
| | C/A | 35 (45.5%) | 52 (51%) | 0.88 (0.44-1.76) | | | |
| | C/C | 14 (18.2%) | 12 (11.8%) | 0.54 (0.20-1.43) | | | |
| Dominant | A/A | 28 (36.4%) | 38 (37.2%) | 1.00 | 0.47 | 233.3 | 246 |
| | C/A-C/C | 49 (63.6%) | 64 (62.8%) | 0.79 (0.41-1.52) | | | |
| Recessive | A/A-C/A | 63 (81.8%) | 90 (88.2%) | 1.00 | 0.23 | 232.3 | 245.1 |
| | C/C | 14 (18.2%) | 12 (11.8%) | 0.58 (0.24-1.41) | | | |
| Overdominant | A/A-C/C | 42 (54.5%) | 50 (49%) | 1.00 | 0.87 | 233.8 | 246.5 |
| | C/A | 35 (45.5%) | 52 (51%) | 1.06 (0.56-1.98) | | | |
| Log-additive | --- | --- | --- | 0.76 (0.48-1.22) | 0.26 | 232.5 | 245.3 |

### Interaction analysis with covariate gender

| rs4256 and gender cross-classification interaction table (n=179, adjusted by age) | | | | | | |
|---|---|---|---|---|---|---|
| | **FeMale** | | | **Male** | | |
| | **status=0-control** | **status=1-cese** | **OR (95% CI)** | **status=0-control** | **status=1-cese** | **OR (95% CI)** |
| **A/A** | 16 | 22 | 1.00 | 12 | 16 | 0.97 (0.34-2.74) |
| **C/A** | 19 | 39 | 1.14 (0.46-2.79) | 16 | 13 | 0.57 (0.21-1.59) |
| **C/C** | 11 | 9 | 0.49 (0.15-1.56) | 3 | 3 | 0.88 (0.15-5.24) |
| **Interaction p-value:** 0.43 | | | | | | |

| gender within rs4256 (n=179, adjusted by age) | | | | |
|---|---|---|---|---|
| | | **status=0-control** | **status=1-cese** | **OR (95% CI)** |
| **A/A** | **FeMale** | 16 | 22 | 1.00 |
| | **Male** | 12 | 16 | 0.97 (0.34-2.74) |
| | | **status=0-control** | **status=1-cese** | **OR (95% CI)** |
| **C/A** | **FeMale** | 19 | 39 | 1.00 |
| | **Male** | 16 | 13 | 0.50 (0.19-1.31) |
| | | **status=0-control** | **status=1-cese** | **OR (95% CI)** |
| **C/C** | **FeMale** | 11 | 9 | 1.00 |
| | **Male** | 3 | 3 | 1.78 (0.26-12.01) |
| **Test for interaction in the trend:** 0.91 | | | | |

| rs4256 within gender (n=179, adjusted by age) | | | | |
|---|---|---|---|---|
| | | **status=0-control** | **status=1-cese** | **OR (95% CI)** |
| **FeMale** | **A/A** | 16 | 22 | 1.00 |
| | **C/A** | 19 | 39 | 1.14 (0.46-2.79) |
| | **C/C** | 11 | 9 | 0.49 (0.15-1.56) |
| | | **status=0-control** | **status=1-cese** | **OR (95% CI)** |
| **Male** | **A/A** | 12 | 16 | 1.00 |
| | **C/A** | 16 | 13 | 0.59 (0.20-1.76) |
| | **C/C** | 3 | 3 | 0.90 (0.14-5.64) |
| **Test for interaction in the trend:** 0.43 | | | | |

---

## SNP: rs0519

**Percentage of typed samples:** 179/179 (100%)

| rs0519 allele frequencies (n=179) |
|---|

| | All subjects | | status=0-control | | status=1-cese | |
|---|---|---|---|---|---|---|
| **Allele** | **Count** | **Proportion** | **Count** | **Proportion** | **Count** | **Proportion** |
| G | 244 | 0.68 | 105 | 0.68 | 139 | 0.68 |
| A | 114 | 0.32 | 49 | 0.32 | 65 | 0.32 |

| rs0519 genotype frequencies (n=179) | | | | | | |
|---|---|---|---|---|---|---|
| | **All subjects** | | **status=0-control** | | **status=1-cese** | |
| **Genotype** | **Count** | **Proportion** | **Count** | **Proportion** | **Count** | **Proportion** |
| A/A | 14 | 0.08 | 6 | 0.08 | 8 | 0.08 |
| G/A | 86 | 0.48 | 37 | 0.48 | 49 | 0.48 |
| G/G | 79 | 0.44 | 34 | 0.44 | 45 | 0.44 |

| rs0519 exact test for Hardy-Weinberg equilibrium (n=179) | | | | | | |
|---|---|---|---|---|---|---|
| | **N11** | **N12** | **N22** | **N1** | **N2** | **P-value** |
| **All subjects** | 79 | 86 | 14 | 244 | 114 | 0.17 |
| **status=0-control** | 34 | 37 | 6 | 105 | 49 | 0.44 |
| **status=1-cese** | 45 | 49 | 8 | 139 | 65 | 0.36 |

**rs0519 association with response status (n=179, adjusted by age+gender)**

| Model | Genotype | status=0-control | status=1-cese | OR (95% CI) | P-value | AIC | BIC |
|---|---|---|---|---|---|---|---|
| Codominant | G/G | 34 (44.2%) | 45 (44.1%) | 1.00 | 0.82 | 235.4 | 251.4 |
| | A/G | 37 (48%) | 49 (48%) | 0.82 (0.42-1.59) | | | |
| | A/A | 6 (7.8%) | 8 (7.8%) | 0.80 (0.23-2.71) | | | |
| Dominant | G/G | 34 (44.2%) | 45 (44.1%) | 1.00 | 0.54 | 233.4 | 246.2 |
| | A/G-A/A | 43 (55.8%) | 57 (55.9%) | 0.82 (0.43-1.55) | | | |
| Recessive | G/G-A/G | 71 (92.2%) | 94 (92.2%) | 1.00 | 0.84 | 233.8 | 246.5 |
| | A/A | 6 (7.8%) | 8 (7.8%) | 0.89 (0.27-2.85) | | | |
| Overdominant | G/G-A/A | 40 (52%) | 53 (52%) | 1.00 | 0.61 | 233.5 | 246.3 |
| | A/G | 37 (48%) | 49 (48%) | 0.85 (0.45-1.60) | | | |
| Log-additive | --- | --- | --- | 0.86 (0.52-1.43) | 0.56 | 233.5 | 246.2 |

## Interaction analysis with covariate gender

| rs0519 and gender cross-classification interaction table (n=179, adjusted by age) | | | | | | |
|---|---|---|---|---|---|---|
| | **FeMale** | | | **Male** | | |
| | **status=0-control** | **status=1-cese** | **OR (95% CI)** | **status=0-control** | **status=1-cese** | **OR (95% CI)** |
| **G/G** | 20 | 27 | 1.00 | 14 | 18 | 0.97 (0.37-2.51) |
| **A/G** | 21 | 37 | 1.01 (0.44-2.33) | 16 | 12 | 0.56 (0.21-1.51) |
| **A/A** | 5 | 6 | 0.67 (0.16-2.70) | 1 | 2 | 1.77 (0.14-23.01) |
| **Interaction p-value:** 0.48 | | | | | | |

| gender within rs0519 (n=179, adjusted by age) | | | | |
|---|---|---|---|---|
| | | **status=0-control** | **status=1-cese** | **OR (95% CI)** |
| **G/G** | **FeMale** | 20 | 27 | 1.00 |
| | **Male** | 14 | 18 | 0.97 (0.37-2.51) |
| | | **status=0-control** | **status=1-cese** | **OR (95% CI)** |
| **A/G** | **FeMale** | 21 | 37 | 1.00 |
| | **Male** | 16 | 12 | 0.55 (0.21-1.45) |
| | | **status=0-control** | **status=1-cese** | **OR (95% CI)** |
| **A/A** | **FeMale** | 5 | 6 | 1.00 |
| | **Male** | 1 | 2 | 2.65 (0.16-43.48) |
| **Test for interaction in the trend:** 0.88 | | | | |

| rs0519 within gender (n=179, adjusted by age) | | | | |
|---|---|---|---|---|
| **FeMale** | | **status=0-control** | **status=1-cese** | **OR (95% CI)** |
| | **G/G** | 20 | 27 | 1.00 |

| | | | | |
|---|---|---|---|---|
| | **A/G** | 21 | 37 | 1.01 (0.44-2.33) |
| | **A/A** | 5 | 6 | 0.67 (0.16-2.70) |
| **Male** | | **status=0-control** | **status=1-cese** | **OR (95% CI)** |
| | **G/G** | 14 | 18 | 1.00 |
| | **A/G** | 16 | 12 | 0.58 (0.20-1.68) |
| | **A/A** | 1 | 2 | 1.83 (0.14-24.56) |
| **Test for interaction in the trend:** 0.48 | | | | |

---

## SNP: rs0270

**Percentage of typed samples:** 179/179 (100%)

| rs0270 allele frequencies (n=179) | | | | | | |
|---|---|---|---|---|---|---|
| | All subjects | | status=0-control | | status=1-cese | |
| **Allele** | **Count** | **Proportion** | **Count** | **Proportion** | **Count** | **Proportion** |
| A | 202 | 0.56 | 85 | 0.55 | 117 | 0.57 |
| G | 156 | 0.44 | 69 | 0.45 | 87 | 0.43 |

| rs0270 genotype frequencies (n=179) | | | | | | |
|---|---|---|---|---|---|---|
| | All subjects | | status=0-control | | status=1-cese | |
| **Genotype** | **Count** | **Proportion** | **Count** | **Proportion** | **Count** | **Proportion** |
| A/A | 56 | 0.31 | 23 | 0.3 | 33 | 0.32 |
| A/G | 90 | 0.5 | 39 | 0.51 | 51 | 0.5 |
| G/G | 33 | 0.18 | 15 | 0.19 | 18 | 0.18 |

| rs0270 exact test for Hardy-Weinberg equilibrium (n=179) | | | | | | |
|---|---|---|---|---|---|---|
| | **N11** | **N12** | **N22** | **N1** | **N2** | **P-value** |
| **All subjects** | 56 | 90 | 33 | 202 | 156 | 0.88 |
| **status=0-control** | 23 | 39 | 15 | 85 | 69 | 1 |
| **status=1-cese** | 33 | 51 | 18 | 117 | 87 | 1 |

| rs0270 association with response status (n=179, adjusted by age+gender) | | | | | | | |
|---|---|---|---|---|---|---|---|
| **Model** | **Genotype** | **status=0-control** | **status=1-cese** | **OR (95% CI)** | **P-value** | **AIC** | **BIC** |
| Codominant | A/A | 23 (29.9%) | 33 (32.4%) | 1.00 | 0.48 | 234.3 | 250.3 |
| | G/A | 39 (50.6%) | 51 (50%) | 0.66 (0.32-1.38) | | | |
| | G/G | 15 (19.5%) | 18 (17.6%) | 0.64 (0.25-1.62) | | | |
| Dominant | A/A | 23 (29.9%) | 33 (32.4%) | 1.00 | 0.23 | 232.4 | 245.1 |
| | G/A-G/G | 54 (70.1%) | 69 (67.7%) | 0.65 (0.33-1.31) | | | |
| Recessive | A/A-G/A | 62 (80.5%) | 84 (82.3%) | 1.00 | 0.64 | 233.6 | 246.3 |
| | G/G | 15 (19.5%) | 18 (17.6%) | 0.82 (0.36-1.85) | | | |
| Overdominant | A/A-G/G | 38 (49.4%) | 51 (50%) | 1.00 | 0.45 | 233.2 | 246 |
| | G/A | 39 (50.6%) | 51 (50%) | 0.78 (0.41-1.48) | | | |
| Log-additive | --- | --- | --- | 0.78 (0.49-1.23) | 0.29 | 232.7 | 245.4 |

**Interaction analysis with covariate gender**

| rs0270 and gender cross-classification interaction table (n=179, adjusted by age) | | | | | | |
|---|---|---|---|---|---|---|
| | FeMale | | | Male | | |
| | **status=0-control** | **status=1-cese** | **OR (95% CI)** | **status=0-control** | **status=1-cese** | **OR (95% CI)** |
| **A/A** | 13 | 20 | 1.00 | 10 | 13 | 0.83 (0.26-2.60) |
| **G/A** | 21 | 36 | 0.74 (0.29-1.93) | 18 | 15 | 0.45 (0.16-1.27) |
| **G/G** | 12 | 14 | 0.54 (0.17-1.65) | 3 | 4 | 0.98 (0.18-5.42) |
| **Interaction p-value:** 0.54 | | | | | | |

| gender within rs0270 (n=179, adjusted by age) | | | | |
|---|---|---|---|---|
| **A/A** | | **status=0-control** | **status=1-cese** | **OR (95% CI)** |
| | **FeMale** | 13 | 20 | 1.00 |

| | | | | |
|---|---|---|---|---|
| | Male | 10 | 13 | 0.83 (0.26-2.60) |
| G/A | | status=0-control | status=1-cese | OR (95% CI) |
| | FeMale | 21 | 36 | 1.00 |
| | Male | 18 | 15 | 0.60 (0.24-1.51) |
| G/G | | status=0-control | status=1-cese | OR (95% CI) |
| | FeMale | 12 | 14 | 1.00 |
| | Male | 3 | 4 | 1.82 (0.31-10.65) |
| **Test for interaction in the trend:** 0.73 | | | | |

| rs0270 within gender (n=179, adjusted by age) | | | | |
|---|---|---|---|---|
| FeMale | | status=0-control | status=1-cese | OR (95% CI) |
| | A/A | 13 | 20 | 1.00 |
| | G/A | 21 | 36 | 0.74 (0.29-1.93) |
| | G/G | 12 | 14 | 0.54 (0.17-1.65) |
| Male | | status=0-control | status=1-cese | OR (95% CI) |
| | A/A | 10 | 13 | 1.00 |
| | G/A | 18 | 15 | 0.54 (0.17-1.69) |
| | G/G | 3 | 4 | 1.18 (0.20-6.98) |
| **Test for interaction in the trend:** 0.54 | | | | |

---

## Multiple-SNP analysis

### Linkage disequilibrium analysis

### D statistic

| | rs4256 | rs0519 | rs0270 |
|---|---|---|---|
| rs4256 | . | 0.1947 | 0.219 |
| rs0519 | . | . | 0.1796 |
| rs0270 | . | . | . |

### D' statistic

| | rs4256 | rs0519 | rs0270 |
|---|---|---|---|
| rs4256 | . | 0.9998 | 0.9997 |
| rs0519 | . | . | 0.9997 |
| rs0270 | . | . | . |

### r statistic

| | rs4256 | rs0519 | rs0270 |
|---|---|---|---|
| rs4256 | . | 0.8578 | 0.9063 |
| rs0519 | . | . | 0.7776 |
| rs0270 | . | . | . |

### P-values

| | rs4256 | rs0519 | rs0270 |
|---|---|---|---|
| rs4256 | . | 0 | 0 |
| rs0519 | . | . | 0 |
| rs0270 | . | . | . |

### Haplotype analysis

| Haplotype frequencies estimation (n=179) | | | | | | | |
|---|---|---|---|---|---|---|---|
| | rs4256 | rs0519 | rs0270 | Total | group.0.control | group.1.cese | Cumulative frequency |
| 1 | A | G | A | 0.5642 | 0.5519 | 0.5735 | 0.5642 |
| 2 | C | A | G | 0.3184 | 0.3182 | 0.3186 | 0.8827 |
| 3 | C | G | G | 0.0698 | 0.0909 | 0.0539 | 0.9525 |
| 4 | A | G | G | 0.0475 | 0.039 | 0.0539 | 1 |

**Haplotype association with response (n=179, adjusted by age+gender)**

| | rs4256 | rs0519 | rs0270 | Freq | OR (95% CI) | P-value |
|---|---|---|---|---|---|---|
| 1 | A | G | A | 0.5642 | 1.00 | --- |
| 2 | C | A | G | 0.3184 | 0.83 (0.49 - 1.41) | 0.49 |
| 3 | C | G | G | 0.0698 | 0.56 (0.23 - 1.40) | 0.22 |
| 4 | A | G | G | 0.0475 | 1.00 (0.32 - 3.10) | 1 |
| **Global haplotype association p-value:** 0.59 | | | | | | |

### Haplotype interaction analysis with covariate gender

| **Haplotype and gender cross-classification interaction table (n=179, adjusted by age)** | | | |
|---|---|---|---|
| | | **FeMale** | **Male** |
| **Haplotype** | **Frequency** | **OR (95% CI)** | **OR (95% CI)** |
| **AGA** | 0.5642 | 1.00 | 0.68 (0.24 - 1.90) |
| **CAG** | 0.3184 | 0.83 (0.44 - 1.58) | 0.57 (0.22 - 1.46) |
| **AGG** | 0.0475 | 0.80 (0.19 - 3.35) | 1.01 (0.16 - 6.41) |
| **CGG** | 0.0698 | 0.50 (0.17 - 1.48) | 0.54 (0.10 - 3.00) |
| **Interaction p-value:** 0.93 | | | |

| **Haplotypes within gender (n=179, adjusted by age)** | | | |
|---|---|---|---|
| | | **FeMale** | **Male** |
| **Haplotype** | **Frequency** | **OR (95% CI)** | **OR (95% CI)** |
| **AGA** | 0.5642 | 1.00 | 1.00 |
| **CAG** | 0.3184 | 0.83 (0.44 - 1.58) | 0.84 (0.34 - 2.08) |
| **AGG** | 0.0475 | 0.80 (0.19 - 3.35) | 1.49 (0.23 - 9.53) |
| **CGG** | 0.0698 | 0.50 (0.17 - 1.48) | 0.79 (0.15 - 4.18) |

| **gender whithin haplotypes (n=179, adjusted by age)** | | | |
|---|---|---|---|
| | | **FeMale** | **Male** |
| **Haplotype** | **Frequency** | **OR (95% CI)** | **OR (95% CI)** |
| **AGA** | 0.5642 | 1.00 | 0.68 (0.24 - 1.90) |
| **CAG** | 0.3184 | 1.00 | 0.68 (0.30 - 1.57) |
| **AGG** | 0.0475 | 1.00 | 1.25 (0.14 - 11.36) |
| **CGG** | 0.0698 | 1.00 | 1.08 (0.16 - 7.31) |

<<< Step 3: Customize analysis
