# Supplementary material for: Association of tyrosine kinase 2 polymorphisms with susceptibility to microscopic polyangiitis in a Guangxi population
Source: PeerJ. 2024 Dec 23;12:e18735. doi: 10.7717/peerj.18735 (PMC11670758; doi:10.7717/peerj.18735)
Supplement: Supplemental Information 10 [file peerj-12-18735-s010.pdf]

SNPStats results

Index

Descriptive statistics
Single-SNP analysis
- rs4256
- rs0519
- rs0270

Multiple-SNP analysis
- Linkage disequilibrium analysis
- Haplotype analysis

Descriptive statistics

Response variable: status Type: categorical

	n	missing	unique
**All subjects**	182	0	2
**status=0-control**	53 (29.12%)	---	---
**status=1-cese**	129 (70.88%)	---	---

Covariate: ethnicity Type: categorical

	n	missing	unique
**All subjects**	182	0	2
**status=0-control**	53	0	2
**status=1-cese**	129	0	2

	1	2
**All subjects**	132 (73%)	50 (27%)
**status=0-control**	48 (91%)	5 (9%)
**status=1-cese**	84 (65%)	45 (35%)

Covariate: gender Type: categorical

	n	missing	unique
**All subjects**	182	0	2
**status=0-control**	53	0	2
**status=1-cese**	129	0	2

	FeMale	Male
**All subjects**	113 (62%)	69 (38%)
**status=0-control**	29 (55%)	24 (45%)
**status=1-cese**	84 (65%)	45 (35%)

Single-SNP analysis

SNP: rs4256

Percentage of typed samples: 182/182 (100%)

rs4256 allele frequencies (n=182)						
	All subjects		status=0-control		status=1-cese	
**Allele**	**Count**	**Proportion**	**Count**	**Proportion**	**Count**	**Proportion**
A	223	0.61	69	0.65	154	0.6
C	141	0.39	37	0.35	104	0.4

rs4256 genotype frequencies (n=182)						
	All subjects		status=0-control		status=1-cese	
**Genotype**	**Count**	**Proportion**	**Count**	**Proportion**	**Count**	**Proportion**

A/A	66	0.36	21	0.4	45	0.35
A/C	91	0.5	27	0.51	64	0.5
C/C	25	0.14	5	0.09	20	0.16

rs4256 exact test for Hardy-Weinberg equilibrium (n=182)						
	**N11**	**N12**	**N22**	**N1**	**N2**	**P-value**
**All subjects**	66	91	25	223	141	0.53
**status=0-control**	21	27	5	69	37	0.55
**status=1-cese**	45	64	20	154	104	0.85

rs4256 association with response status (n=182, adjusted by ethnicity+gender)							
**Model**	**Genotype**	**status=0-control**	**status=1-cese**	**OR (95% CI)**	**P-value**	**AIC**	**BIC**
Codominant	A/A	21 (39.6%)	45 (34.9%)	1.00	0.53	214	230
	C/A	27 (50.9%)	64 (49.6%)	0.98 (0.48-2.00)			
	C/C	5 (9.4%)	20 (15.5%)	1.78 (0.57-5.57)			
Dominant	A/A	21 (39.6%)	45 (34.9%)	1.00	0.78	213.2	226
	C/A-C/C	32 (60.4%)	84 (65.1%)	1.10 (0.56-2.19)			
Recessive	A/A-C/A	48 (90.6%)	109 (84.5%)	1.00	0.26	212	224.8
	C/C	5 (9.4%)	20 (15.5%)	1.81 (0.62-5.24)			
Overdominant	A/A-C/C	26 (49.1%)	65 (50.4%)	1.00	0.62	213	225.8
	C/A	27 (50.9%)	64 (49.6%)	0.85 (0.43-1.65)			
Log-additive	---	---	---	1.21 (0.74-2.00)	0.44	212.7	225.5

Interaction analysis with covariate ethnicity

rs4256 and ethnicity cross-classification interaction table (n=182, adjusted by gender)						
	**1**			**2**		
	**status=0-control**	**status=1-cese**	**OR (95% CI)**	**status=0-control**	**status=1-cese**	**OR (95% CI)**
**A/A**	20	31	1.00	1	14	**8.64 (1.05-71.34)**
**C/A**	24	39	1.03 (0.48-2.20)	3	25	**5.05 (1.32-19.32)**
**C/C**	4	14	2.23 (0.64-7.77)	1	6	3.61 (0.40-32.74)
**Interaction p-value:** 0.6						

ethnicity within rs4256 (n=182, adjusted by gender)				
		**status=0-control**	**status=1-cese**	**OR (95% CI)**
**A/A**	**1**	20	31	1.00
	**2**	1	14	**8.64 (1.05-71.34)**
		**status=0-control**	**status=1-cese**	**OR (95% CI)**
**C/A**	**1**	24	39	1.00
	**2**	3	25	**4.92 (1.33-18.23)**
		**status=0-control**	**status=1-cese**	**OR (95% CI)**
**C/C**	**1**	4	14	1.00
	**2**	1	6	1.62 (0.15-17.84)
**Test for interaction in the trend:** 0.36				

rs4256 within ethnicity (n=182, adjusted by gender)				
		**status=0-control**	**status=1-cese**	**OR (95% CI)**
**1**	**A/A**	20	31	1.00
	**C/A**	24	39	1.03 (0.48-2.20)
	**C/C**	4	14	2.23 (0.64-7.77)
		**status=0-control**	**status=1-cese**	**OR (95% CI)**
**2**	**A/A**	1	14	1.00
	**C/A**	3	25	0.58 (0.06-6.18)
	**C/C**	1	6	0.42 (0.02-7.86)
**Test for interaction in the trend:** 0.6				

SNP: rs0519

Percentage of typed samples: 182/182 (100%)

rs0519 allele frequencies (n=182)						
	All subjects		status=0-control		status=1-cese	
**Allele**	**Count**	**Proportion**	**Count**	**Proportion**	**Count**	**Proportion**
G	240	0.66	74	0.7	166	0.64
A	124	0.34	32	0.3	92	0.36

rs0519 genotype frequencies (n=182)						
	All subjects		status=0-control		status=1-cese	
**Genotype**	**Count**	**Proportion**	**Count**	**Proportion**	**Count**	**Proportion**
A/A	20	0.11	4	0.08	16	0.12
G/A	84	0.46	24	0.45	60	0.47
G/G	78	0.43	25	0.47	53	0.41

rs0519 exact test for Hardy-Weinberg equilibrium (n=182)						
	**N11**	**N12**	**N22**	**N1**	**N2**	**P-value**
**All subjects**	78	84	20	240	124	0.87
**status=0-control**	25	24	4	74	32	0.75
**status=1-cese**	53	60	16	166	92	1

rs0519 association with response status (n=182, adjusted by ethnicity+gender)							
**Model**	**Genotype**	**status=0-control**	**status=1-cese**	**OR (95% CI)**	**P-value**	**AIC**	**BIC**
Codominant	G/G	25 (47.2%)	53 (41.1%)	1.00	0.55	214.1	230.1
	A/G	24 (45.3%)	60 (46.5%)	1.08 (0.54-2.17)			
	A/A	4 (7.5%)	16 (12.4%)	1.92 (0.56-6.52)			
Dominant	G/G	25 (47.2%)	53 (41.1%)	1.00	0.6	213	225.8
	A/G-A/A	28 (52.8%)	76 (58.9%)	1.20 (0.61-2.34)			
Recessive	G/G-A/G	49 (92.5%)	113 (87.6%)	1.00	0.29	212.1	224.9
	A/A	4 (7.5%)	16 (12.4%)	1.84 (0.57-5.96)			
Overdominant	G/G-A/A	29 (54.7%)	69 (53.5%)	1.00	0.9	213.3	226.1
	A/G	24 (45.3%)	60 (46.5%)	0.96 (0.49-1.87)			
Log-additive	---	---	---	1.26 (0.76-2.09)	0.37	212.5	225.3

Interaction analysis with covariate ethnicity

rs0519 and ethnicity cross-classification interaction table (n=182, adjusted by gender)						
	1			2		
	**status=0-control**	**status=1-cese**	**OR (95% CI)**	**status=0-control**	**status=1-cese**	**OR (95% CI)**
**G/G**	23	36	1.00	2	17	**5.24 (1.10-24.99)**
**A/G**	21	37	1.12 (0.53-2.36)	3	23	**4.63 (1.23-17.51)**
**A/A**	4	11	1.74 (0.49-6.12)	0	5	---
**Interaction p-value:** 0.7						

ethnicity within rs0519 (n=182, adjusted by gender)				
		**status=0-control**	**status=1-cese**	**OR (95% CI)**
**G/G**	**1**	23	36	1.00
	**2**	2	17	**5.24 (1.10-24.99)**
		**status=0-control**	**status=1-cese**	**OR (95% CI)**
**A/G**	**1**	21	37	1.00
	**2**	3	23	**4.15 (1.10-15.68)**
		**status=0-control**	**status=1-cese**	**OR (95% CI)**
**A/A**	**1**	4	11	1.00
	**2**	0	5	---
**Test for interaction in the trend:** 0.89				

rs0519 within ethnicity (n=182, adjusted by gender)				
		status=0-control	status=1-cese	OR (95% CI)
1	G/G	23	36	1.00
	A/G	21	37	1.12 (0.53-2.36)
	A/A	4	11	1.74 (0.49-6.12)
		status=0-control	status=1-cese	OR (95% CI)
2	G/G	2	17	1.00
	A/G	3	23	0.88 (0.13-5.90)
	A/A	0	5	---
**Test for interaction in the trend:** 0.7				

SNP: rs0270

Percentage of typed samples: 182/182 (100%)

rs0270 allele frequencies (n=182)						
	All subjects		status=0-control		status=1-cese	
**Allele**	**Count**	**Proportion**	**Count**	**Proportion**	**Count**	**Proportion**
A	208	0.57	65	0.61	143	0.55
G	156	0.43	41	0.39	115	0.45

rs0270 genotype frequencies (n=182)						
	All subjects		status=0-control		status=1-cese	
**Genotype**	**Count**	**Proportion**	**Count**	**Proportion**	**Count**	**Proportion**
A/A	56	0.31	18	0.34	38	0.29
A/G	96	0.53	29	0.55	67	0.52
G/G	30	0.16	6	0.11	24	0.19

rs0270 exact test for Hardy-Weinberg equilibrium (n=182)						
	**N11**	**N12**	**N22**	**N1**	**N2**	**P-value**
**All subjects**	56	96	30	208	156	0.36
**status=0-control**	18	29	6	65	41	0.39
**status=1-cese**	38	67	24	143	115	0.6

rs0270 association with response status (n=182, adjusted by ethnicity+gender)							
**Model**	**Genotype**	**status=0-control**	**status=1-cese**	**OR (95% CI)**	**P-value**	**AIC**	**BIC**
Codominant	A/A	18 (34%)	38 (29.5%)	1.00	0.63	214.4	230.4
	G/A	29 (54.7%)	67 (51.9%)	0.95 (0.45-1.98)			
	G/G	6 (11.3%)	24 (18.6%)	1.54 (0.52-4.60)			
Dominant	A/A	18 (34%)	38 (29.5%)	1.00	0.9	213.3	226.1
	G/A-G/G	35 (66%)	91 (70.5%)	1.05 (0.51-2.14)			
Recessive	A/A-G/A	47 (88.7%)	105 (81.4%)	1.00	0.34	212.4	225.2
	G/G	6 (11.3%)	24 (18.6%)	1.59 (0.59-4.29)			
Overdominant	A/A-G/G	24 (45.3%)	62 (48.1%)	1.00	0.58	213	225.8
	G/A	29 (54.7%)	67 (51.9%)	0.83 (0.43-1.62)			
Log-additive	---	---	---	1.16 (0.70-1.92)	0.55	212.9	225.7

Interaction analysis with covariate ethnicity

rs0270 and ethnicity cross-classification interaction table (n=182, adjusted by gender)						
	1			2		
	status=0-control	status=1-cese	OR (95% CI)	status=0-control	status=1-cese	OR (95% CI)
**A/A**	18	27	1.00	0	11	---
**G/A**	25	43	1.12 (0.51-2.44)	4	24	**3.79 (1.10-13.00)**
**G/G**	5	14	1.83 (0.56-5.99)	1	10	6.17 (0.71-53.60)
**Interaction p-value:** 0.22						

ethnicity within rs0270 (n=182, adjusted by gender)				
		status=0-control	status=1-cese	OR (95% CI)
A/A	1	18	27	1.00
	2	0	11	---
		status=0-control	status=1-cese	OR (95% CI)
G/A	1	25	43	1.00
	2	4	24	**3.38 (1.04-10.94)**
		status=0-control	status=1-cese	OR (95% CI)
G/G	1	5	14	1.00
	2	1	10	3.37 (0.34-33.84)
**Test for interaction in the trend:** 0.31				

rs0270 within ethnicity (n=182, adjusted by gender)				
		status=0-control	status=1-cese	OR (95% CI)
1	A/A	18	27	1.00
	G/A	25	43	1.12 (0.51-2.44)
	G/G	5	14	1.83 (0.56-5.99)
		status=0-control	status=1-cese	OR (95% CI)
2	A/A	0	11	1.00
	G/A	4	24	0.00
	G/G	1	10	0.00
**Test for interaction in the trend:** 0.22				

Multiple-SNP analysis

Linkage disequilibrium analysis

D statistic

	rs4256	rs0519	rs0270
rs4256	.	0.2086	0.2213
rs0519	.	.	0.1946
rs0270	.	.	.

D' statistic

	rs4256	rs0519	rs0270
rs4256	.	0.9997	0.9997
rs0519	.	.	0.9997
rs0270	.	.	.

r statistic

	rs4256	rs0519	rs0270
rs4256	.	0.9037	0.9179
rs0519	.	.	0.8298
rs0270	.	.	.

P-values

	rs4256	rs0519	rs0270
rs4256	.	0	0
rs0519	.	.	0
rs0270	.	.	.

Haplotype analysis

Haplotype frequencies estimation (n=182)							
	rs4256	rs0519	rs0270	Total	group.0.control	group.1.cese	Cumulative frequency
1	A	G	A	0.5714	0.6132	0.5543	0.5714
2	C	A	G	0.3407	0.3019	0.3566	0.9121

3	C	G	G	0.0467	0.0472	0.0465	0.9588
4	A	G	G	0.0412	0.0377	0.0426	1

**Haplotype association with response (n=182, adjusted by ethnicity+gender)**						
	**rs4256**	**rs0519**	**rs0270**	**Freq**	**OR (95% CI)**	**P-value**
1	A	G	A	0.5714	1.00	---
2	C	A	G	0.3407	1.23 (0.73 - 2.08)	0.45
3	C	G	G	0.0467	0.91 (0.28 - 2.98)	0.88
4	A	G	G	0.0412	0.79 (0.21 - 2.98)	0.73
**Global haplotype association p-value:** 0.82						

Haplotype interaction analysis with covariate ethnicity

**Haplotype and ethnicity cross-classification interaction table (n=182, adjusted by gender)**			
		**1**	**2**
**Haplotype**	**Frequency**	**OR (95% CI)**	**OR (95% CI)**
**AGA**	0.5714	1.00	**12.88 (1.49 - 111.09)**
**CAG**	0.3407	1.27 (0.73 - 2.23)	**11.08 (2.34 - 52.40)**
**AGG**	0.0412	0.87 (0.18 - 4.24)	4.94 (0.47 - 51.86)
**CGG**	0.0467	1.71 (0.42 - 6.97)	1.38 (0.21 - 9.16)
**Interaction p-value:** 0.28			

**Haplotypes within ethnicity (n=182, adjusted by gender)**			
		**1**	**2**
**Haplotype**	**Frequency**	**OR (95% CI)**	**OR (95% CI)**
**AGA**	0.5714	1.00	1.00
**CAG**	0.3407	1.27 (0.73 - 2.23)	0.86 (0.16 - 4.76)
**AGG**	0.0412	0.87 (0.18 - 4.24)	0.38 (0.03 - 5.23)
**CGG**	0.0467	1.71 (0.42 - 6.97)	0.11 (0.01 - 1.19)

**ethnicity whithin haplotypes (n=182, adjusted by gender)**			
		**1**	**2**
**Haplotype**	**Frequency**	**OR (95% CI)**	**OR (95% CI)**
**AGA**	0.5714	1.00	**12.88 (1.49 - 111.09)**
**CAG**	0.3407	1.00	**8.70 (1.93 - 39.25)**
**AGG**	0.0412	1.00	5.70 (0.37 - 87.19)
**CGG**	0.0467	1.00	0.81 (0.09 - 7.64)

<<< Step 3: Customize analysis
