# Supplementary material for: Association of tyrosine kinase 2 polymorphisms with susceptibility to microscopic polyangiitis in a Guangxi population
Source: PeerJ. 2024 Dec 23;12:e18735. doi: 10.7717/peerj.18735 (PMC11670758; doi:10.7717/peerj.18735)
Supplement: Supplemental Information 11 [file peerj-12-18735-s011.pdf]

SNPStats results

Index

Descriptive statistics
Single-SNP analysis
- rs4256
- rs0519
- rs0270

Multiple-SNP analysis
- Linkage disequilibrium analysis
- Haplotype analysis

Descriptive statistics

Response variable: status Type: categorical

	n	missing	unique
**All subjects**	380	0	2
**status=0-control**	244 (64.21%)	---	---
**status=1-cese**	136 (35.79%)	---	---

Covariate: ethnicity Type: categorical

	n	missing	unique
**All subjects**	380	0	2
**status=0-control**	244	0	2
**status=1-cese**	136	0	2

	1	2
**All subjects**	251 (66%)	129 (34%)
**status=0-control**	172 (70%)	72 (30%)
**status=1-cese**	79 (58%)	57 (42%)

Covariate: gender Type: categorical

	n	missing	unique
**All subjects**	380	0	2
**status=0-control**	244	0	2
**status=1-cese**	136	0	2

	FeMale	Male
**All subjects**	229 (60%)	151 (40%)
**status=0-control**	144 (59%)	100 (41%)
**status=1-cese**	85 (62%)	51 (38%)

Single-SNP analysis

SNP: rs4256

Percentage of typed samples: 380/380 (100%)

rs4256 allele frequencies (n=380)						
	All subjects		status=0-control		status=1-cese	
**Allele**	**Count**	**Proportion**	**Count**	**Proportion**	**Count**	**Proportion**
A	458	0.6	286	0.59	172	0.63
C	302	0.4	202	0.41	100	0.37

rs4256 genotype frequencies (n=380)						
	All subjects		status=0-control		status=1-cese	
**Genotype**	**Count**	**Proportion**	**Count**	**Proportion**	**Count**	**Proportion**

A/A	135	0.36	82	0.34	53	0.39
A/C	188	0.49	122	0.5	66	0.49
C/C	57	0.15	40	0.16	17	0.12

**rs4256 exact test for Hardy-Weinberg equilibrium (n=380)**						
	**N11**	**N12**	**N22**	**N1**	**N2**	**P-value**
**All subjects**	135	188	57	458	302	0.59
**status=0-control**	82	122	40	286	202	0.69
**status=1-cese**	53	66	17	172	100	0.71

**rs4256 association with response status (n=380, adjusted by ethnicity+gender)**							
**Model**	**Genotype**	**status=0-control**	**status=1-cese**	**OR (95% CI)**	**P-value**	**AIC**	**BIC**
Codominant	A/A	82 (33.6%)	53 (39%)	1.00	0.51	497.9	517.6
	C/A	122 (50%)	66 (48.5%)	0.87 (0.55-1.38)			
	C/C	40 (16.4%)	17 (12.5%)	0.68 (0.35-1.32)			
Dominant	A/A	82 (33.6%)	53 (39%)	1.00	0.38	496.5	512.2
	C/A-C/C	162 (66.4%)	83 (61%)	0.82 (0.53-1.28)			
Recessive	A/A-C/A	204 (83.6%)	119 (87.5%)	1.00	0.32	496.2	512
	C/C	40 (16.4%)	17 (12.5%)	0.73 (0.40-1.36)			
Overdominant	A/A-C/C	122 (50%)	70 (51.5%)	1.00	0.89	497.2	513
	C/A	122 (50%)	66 (48.5%)	0.97 (0.64-1.48)			
Log-additive	---	---	---	0.83 (0.61-1.14)	0.26	495.9	511.7

Interaction analysis with covariate ethnicity

**rs4256 and ethnicity cross-classification interaction table (n=380, adjusted by gender)**						
	**1**			**2**		
	**status=0-control**	**status=1-cese**	**OR (95% CI)**	**status=0-control**	**status=1-cese**	**OR (95% CI)**
**A/A**	55	29	1.00	27	24	1.74 (0.85-3.55)
**C/A**	90	39	0.84 (0.46-1.51)	32	27	1.63 (0.82-3.23)
**C/C**	27	11	0.81 (0.35-1.87)	13	6	0.87 (0.30-2.53)
**Interaction p-value:** 0.69						

**ethnicity within rs4256 (n=380, adjusted by gender)**				
**A/A**		**status=0-control**	**status=1-cese**	**OR (95% CI)**
	**1**	55	29	1.00
	**2**	27	24	1.74 (0.85-3.55)
**C/A**		**status=0-control**	**status=1-cese**	**OR (95% CI)**
	**1**	90	39	1.00
	**2**	32	27	**1.95 (1.03-3.68)**
**C/C**		**status=0-control**	**status=1-cese**	**OR (95% CI)**
	**1**	27	11	1.00
	**2**	13	6	1.08 (0.32-3.59)
**Test for interaction in the trend:** 0.66				

**rs4256 within ethnicity (n=380, adjusted by gender)**				
**1**		**status=0-control**	**status=1-cese**	**OR (95% CI)**
	**A/A**	55	29	1.00
	**C/A**	90	39	0.84 (0.46-1.51)
	**C/C**	27	11	0.81 (0.35-1.87)
**2**		**status=0-control**	**status=1-cese**	**OR (95% CI)**
	**A/A**	27	24	1.00
	**C/A**	32	27	0.94 (0.44-1.99)
	**C/C**	13	6	0.50 (0.16-1.53)
**Test for interaction in the trend:** 0.69				

SNP: rs0519
Percentage of typed samples: 380/380 (100%)

rs0519 allele frequencies (n=380)						
	All subjects		status=0-control		status=1-cese	
**Allele**	**Count**	**Proportion**	**Count**	**Proportion**	**Count**	**Proportion**
G	511	0.67	322	0.66	189	0.69
A	249	0.33	166	0.34	83	0.31

rs0519 genotype frequencies (n=380)						
	All subjects		status=0-control		status=1-cese	
**Genotype**	**Count**	**Proportion**	**Count**	**Proportion**	**Count**	**Proportion**
A/A	37	0.1	24	0.1	13	0.1
G/A	175	0.46	118	0.48	57	0.42
G/G	168	0.44	102	0.42	66	0.49

rs0519 exact test for Hardy-Weinberg equilibrium (n=380)						
	**N11**	**N12**	**N22**	**N1**	**N2**	**P-value**
**All subjects**	168	175	37	511	249	0.42
**status=0-control**	102	118	24	322	166	0.26
**status=1-cese**	66	57	13	189	83	0.84

rs0519 association with response status (n=380, adjusted by ethnicity+gender)							
**Model**	**Genotype**	**status=0-control**	**status=1-cese**	**OR (95% CI)**	**P-value**	**AIC**	**BIC**
Codominant	G/G	102 (41.8%)	66 (48.5%)	1.00	0.47	497.7	517.4
	A/G	118 (48.4%)	57 (41.9%)	0.76 (0.48-1.18)			
	A/A	24 (9.8%)	13 (9.6%)	0.90 (0.42-1.91)			
Dominant	G/G	102 (41.8%)	66 (48.5%)	1.00	0.25	495.9	511.7
	A/G-A/A	142 (58.2%)	70 (51.5%)	0.78 (0.51-1.19)			
Recessive	G/G-A/G	220 (90.2%)	123 (90.4%)	1.00	0.93	497.2	513
	A/A	24 (9.8%)	13 (9.6%)	1.03 (0.50-2.12)			
Overdominant	G/G-A/A	126 (51.6%)	79 (58.1%)	1.00	0.23	495.8	511.6
	A/G	118 (48.4%)	57 (41.9%)	0.77 (0.50-1.18)			
Log-additive	---	---	---	0.87 (0.62-1.21)	0.4	496.5	512.3

Interaction analysis with covariate ethnicity

rs0519 and ethnicity cross-classification interaction table (n=380, adjusted by gender)						
	**1**			**2**		
	**status=0-control**	**status=1-cese**	**OR (95% CI)**	**status=0-control**	**status=1-cese**	**OR (95% CI)**
**G/G**	70	38	1.00	32	28	1.65 (0.86-3.15)
**A/G**	84	31	0.70 (0.39-1.24)	34	26	1.43 (0.75-2.73)
**A/A**	18	10	1.05 (0.44-2.53)	6	3	0.91 (0.22-3.85)
**Interaction p-value:** 0.59						

ethnicity within rs0519 (n=380, adjusted by gender)				
**G/G**		**status=0-control**	**status=1-cese**	**OR (95% CI)**
	**1**	70	38	1.00
	**2**	32	28	1.65 (0.86-3.15)
**A/G**		**status=0-control**	**status=1-cese**	**OR (95% CI)**
	**1**	84	31	1.00
	**2**	34	26	**2.06 (1.07-3.97)**
**A/A**		**status=0-control**	**status=1-cese**	**OR (95% CI)**
	**1**	18	10	1.00
	**2**	6	3	0.86 (0.18-4.25)
**Test for interaction in the trend:** 0.74				

rs0519 within ethnicity (n=380, adjusted by gender)				
		status=0-control	status=1-cese	OR (95% CI)
1	G/G	70	38	1.00
	A/G	84	31	0.70 (0.39-1.24)
	A/A	18	10	1.05 (0.44-2.53)
		status=0-control	status=1-cese	OR (95% CI)
2	G/G	32	28	1.00
	A/G	34	26	0.87 (0.42-1.78)
	A/A	6	3	0.55 (0.13-2.43)
**Test for interaction in the trend:** 0.59				

SNP: rs0270

Percentage of typed samples: 380/380 (100%)

rs0270 allele frequencies (n=380)						
	All subjects		status=0-control		status=1-cese	
Allele	Count	Proportion	Count	Proportion	Count	Proportion
A	433	0.57	271	0.56	162	0.6
G	327	0.43	217	0.44	110	0.4

rs0270 genotype frequencies (n=380)						
	All subjects		status=0-control		status=1-cese	
Genotype	Count	Proportion	Count	Proportion	Count	Proportion
A/A	121	0.32	73	0.3	48	0.35
A/G	191	0.5	125	0.51	66	0.49
G/G	68	0.18	46	0.19	22	0.16

rs0270 exact test for Hardy-Weinberg equilibrium (n=380)						
	N11	N12	N22	N1	N2	P-value
All subjects	121	191	68	433	327	0.68
status=0-control	73	125	46	271	217	0.61
status=1-cese	48	66	22	162	110	1

rs0270 association with response status (n=380, adjusted by ethnicity+gender)							
Model	Genotype	status=0-control	status=1-cese	OR (95% CI)	P-value	AIC	BIC
Codominant	A/A	73 (29.9%)	48 (35.3%)	1.00	0.62	498.3	518
	G/A	125 (51.2%)	66 (48.5%)	0.83 (0.51-1.33)			
	G/G	46 (18.9%)	22 (16.2%)	0.75 (0.40-1.42)			
Dominant	A/A	73 (29.9%)	48 (35.3%)	1.00	0.35	496.4	512.1
	G/A-G/G	171 (70.1%)	88 (64.7%)	0.81 (0.51-1.27)			
Recessive	A/A-G/A	198 (81.2%)	114 (83.8%)	1.00	0.55	496.9	512.6
	G/G	46 (18.9%)	22 (16.2%)	0.84 (0.48-1.48)			
Overdominant	A/A-G/G	119 (48.8%)	70 (51.5%)	1.00	0.67	497.1	512.8
	G/A	125 (51.2%)	66 (48.5%)	0.91 (0.60-1.39)			
Log-additive	---	---	---	0.86 (0.63-1.17)	0.34	496.3	512.1

Interaction analysis with covariate ethnicity

rs0270 and ethnicity cross-classification interaction table (n=380, adjusted by gender)						
	1			2		
	status=0-control	status=1-cese	OR (95% CI)	status=0-control	status=1-cese	OR (95% CI)
A/A	50	26	1.00	23	22	1.88 (0.89-4.01)
G/A	90	39	0.84 (0.46-1.55)	35	27	1.52 (0.76-3.04)
G/G	32	14	0.88 (0.40-1.94)	14	8	1.09 (0.41-2.94)
**Interaction p-value:** 0.81						

ethnicity within rs0270 (n=380, adjusted by gender)				
		**status=0-control**	**status=1-cese**	**OR (95% CI)**
**A/A**	**1**	50	26	1.00
	**2**	23	22	1.88 (0.89-4.01)
		**status=0-control**	**status=1-cese**	**OR (95% CI)**
**G/A**	**1**	90	39	1.00
	**2**	35	27	1.80 (0.96-3.37)
		**status=0-control**	**status=1-cese**	**OR (95% CI)**
**G/G**	**1**	32	14	1.00
	**2**	14	8	1.25 (0.42-3.67)
**Test for interaction in the trend:** 0.58				

rs0270 within ethnicity (n=380, adjusted by gender)				
		**status=0-control**	**status=1-cese**	**OR (95% CI)**
**1**	**A/A**	50	26	1.00
	**G/A**	90	39	0.84 (0.46-1.55)
	**G/G**	32	14	0.88 (0.40-1.94)
		**status=0-control**	**status=1-cese**	**OR (95% CI)**
**2**	**A/A**	23	22	1.00
	**G/A**	35	27	0.80 (0.37-1.74)
	**G/G**	14	8	0.58 (0.20-1.66)
**Test for interaction in the trend:** 0.81				

Multiple-SNP analysis

Linkage disequilibrium analysis

D statistic

	rs4256	rs0519	rs0270
rs4256	.	0.196	0.2263
rs0519	.	.	0.1852
rs0270	.	.	.

D' statistic

	rs4256	rs0519	rs0270
rs4256	.	0.9928	0.9997
rs0519	.	.	0.9921
rs0270	.	.	.

r statistic

	rs4256	rs0519	rs0270
rs4256	.	0.8534	0.9341
rs0519	.	.	0.7969
rs0270	.	.	.

P-values

	rs4256	rs0519	rs0270
rs4256	.	0	0
rs0519	.	.	0
rs0270	.	.	.

Haplotype analysis

Haplotype frequencies estimation (n=380)							
	**rs4256**	**rs0519**	**rs0270**	**Total**	**group.0.control**	**group.1.cese**	**Cumulative frequency**
1	A	G	A	0.5683	0.5553	0.5916	0.5683
2	C	A	G	0.3262	0.3402	0.3012	0.8945

3	C	G	G	0.0712	0.0738	0.0665	0.9657
4	A	G	G	0.0329	0.0307	0.0368	0.9986
5	A	A	A	0.0014	NA	0.004	1

Haplotype association with response (n=380, adjusted by ethnicity+gender)						
	rs4256	rs0519	rs0270	Freq	OR (95% CI)	P-value
1	A	G	A	0.5683	1.00	---
2	C	A	G	0.3262	0.84 (0.59 - 1.18)	0.31
3	C	G	G	0.0712	0.83 (0.45 - 1.55)	0.56
4	A	G	G	0.0329	1.11 (0.50 - 2.43)	0.8
rare	*	*	*	0.0014	**833050856.68 (833050856.63 - 833050856.73)**	<0.0001
**Global haplotype association p-value:** 0.46						

Haplotype interaction analysis with covariate ethnicity

Haplotype and ethnicity cross-classification interaction table (n=380, adjusted by gender)			
		**1**	**2**
**Haplotype**	**Frequency**	**OR (95% CI)**	**OR (95% CI)**
**AGA**	0.5683	1.00	1.97 (0.99 - 3.95)
**CAG**	0.3262	0.87 (0.57 - 1.34)	1.56 (0.84 - 2.89)
**CGG**	0.0712	0.96 (0.44 - 2.12)	1.33 (0.45 - 3.97)
**AGG**	0.0329	1.21 (0.47 - 3.10)	1.86 (0.45 - 7.65)
**rare**	0.0014	Inf	Inf
**Interaction p-value:** 0.98			

Haplotypes within ethnicity (n=380, adjusted by gender)			
		**1**	**2**
**Haplotype**	**Frequency**	**OR (95% CI)**	**OR (95% CI)**
**AGA**	0.5683	1.00	1.00
**CAG**	0.3262	0.87 (0.57 - 1.34)	0.79 (0.44 - 1.42)
**CGG**	0.0712	0.96 (0.44 - 2.12)	0.68 (0.25 - 1.86)
**AGG**	0.0329	1.21 (0.47 - 3.10)	0.94 (0.23 - 3.84)
**rare**	0.0014	Inf	Inf

ethnicity whithin haplotypes (n=380, adjusted by gender)			
		**1**	**2**
**Haplotype**	**Frequency**	**OR (95% CI)**	**OR (95% CI)**
**AGA**	0.5683	1.00	1.97 (0.99 - 3.95)
**CAG**	0.3262	1.00	**1.79 (1.04 - 3.07)**
**CGG**	0.0712	1.00	1.39 (0.40 - 4.80)
**AGG**	0.0329	1.00	1.55 (0.30 - 7.90)
**rare**	0.0014	1.00	0.00 (-Inf - Inf)

<<< Step 3: Customize analysis
